# Supplementary material for: Magnetic Bioprinting and Actuation of Stretchable Muscle Tissue
Source: Adv Healthc Mater. 2025 Oct 12;15(3):e03035. doi: 10.1002/adhm.202503035 (PMC12817118; doi:10.1002/adhm.202503035)
Supplement: Supplementary file 1 — Supporting Information [file ADHM-15-0-s005.pdf]

ADVANCED HEALTHCARE MATERIALS

Supporting Information

for *Adv. Healthcare Mater.*, DOI 10.1002/adhm.202503035

Magnetic Bioprinting and Actuation of Stretchable Muscle Tissue

Noam Demri, Lise Morizur, Simon Dumas, Giacomo Gropplero, Cécile Martinat, Stéphanie Descroix and Claire Wilhelm**

Supporting information

Magnetic bioprinting and actuation of stretchable muscle tissue

Noam Demri[1], Lise Morizur[2], Simon Dumas[1], Giacomo Gropplero[1], Cécile Martinat[2], Stéphanie Descroix[1], and Claire Wilhelm[1]**

1. Laboratoire Physique des cellules et du cancer, PCC, CNRS UMR168, Institut Curie, Sorbonne University, PSL University, 75005 Paris, France.

2. INSERM/UEVE UMR 861, Université Paris Saclay, I-STEM, Corbeil-Essonnes, France

E-mails: noam.demri@curie.fr; claire.wilhelm@curie.fr

Table of contents

Magnetic bioprinting of wrench-shaped 'clip-on' muscle tissues……………………………...2-5
Fig. S1……………………………………………………………………3
Fig. S2…………………………………………………………...………4
Fig. S3..…………………………………………………………...……...5
Trapping the magnetic-printed muscle tissue between two magnetic needles…………………6-11
Fig. S4..…………………………………………………………...……...7
Fig. S5..…………………………………………………………...……...8
Fig. S6..…………………………………………………………...……...9
Fig. S7..…………………………………………………………...……10
Fig. S8..…………………………………………………………...……11
Preserving tissue anisotropy for myogenesis……………………………………………………12-19
Fig. S9..………………………………………………………….………13
Fig. S10…………………………………………………………...……14
Fig. S11…………………………………………………………...……15
Fig. S12…………………………………………………………...……16
Fig. S13…………………………………………………………...……17
Fig. S14………………………………………………………….………18
Fig. S15…………………………………………………………...……19
Stretching improves muscle differentiation…………………………………………….……20-24
Fig. S16…………………………………………………………...……21
Fig. S17………………………………………………………….………22
Fig. S18…………………………………………………………...……23
Fig. S19……………………………………………………………………24
Transposing to myoblasts derived from human induced pluripotent stem cells…….……..…25-37
Fig. S20……………………………………………………………………26
Fig. S21…………………………………………………………...……27
Fig. S22…………………………………………………………...……28
Fig. S23…………………………………………………………...……29
Fig. S24…………………………………………………………...……30
Fig. S25…………………………………………………………...……31
Fig. S26…………………………………………………………...……32
Fig. S27………………………………………………………….………33
Fig. S28…………………………………………………………...……34
Fig. S29……………………………………………………………………35
Fig. S30…………………………………………………………...……36
Fig. S31………………………………………………………….………37
Stretching improves functionality and specialization……………………………….………38-41
Fig. S32……………………………………………………………………39
Fig. S33…………………………………………………………...……40
Fig. S34………………………………………………………….………41
Materials and methods...........……………………………………………………………….42-43
Table S1..………………………………………………………...…43
Table S2..………………………………………………………...…43

Magnetic bioprinting of wrench-shaped 'clip-on' muscle tissues

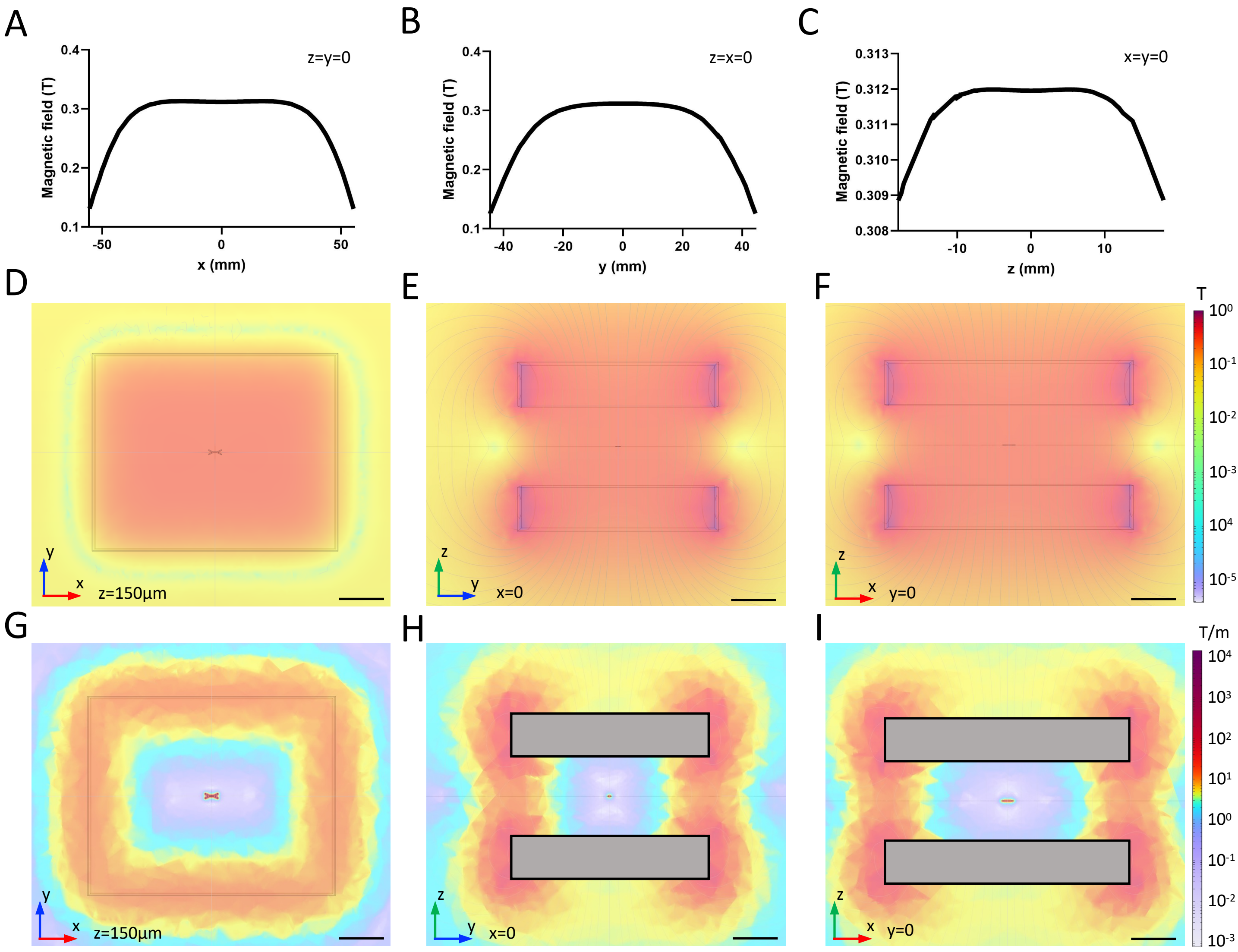

Fig. S1 . (A-C) Graphs of the simulated uniform magnetic field generated at the center the magnets of the magnetic bioprinting set-up without any patterns in it, plotted along the (A) x, (B) y, and (C) z axes. Heatmaps of the magnetic field (D-F) and gradient (G-K) with the field lines generated by the two strong patterns with a single NiFe pattern in the center, visualized in 3 different planes. (Scale bar=2cm)

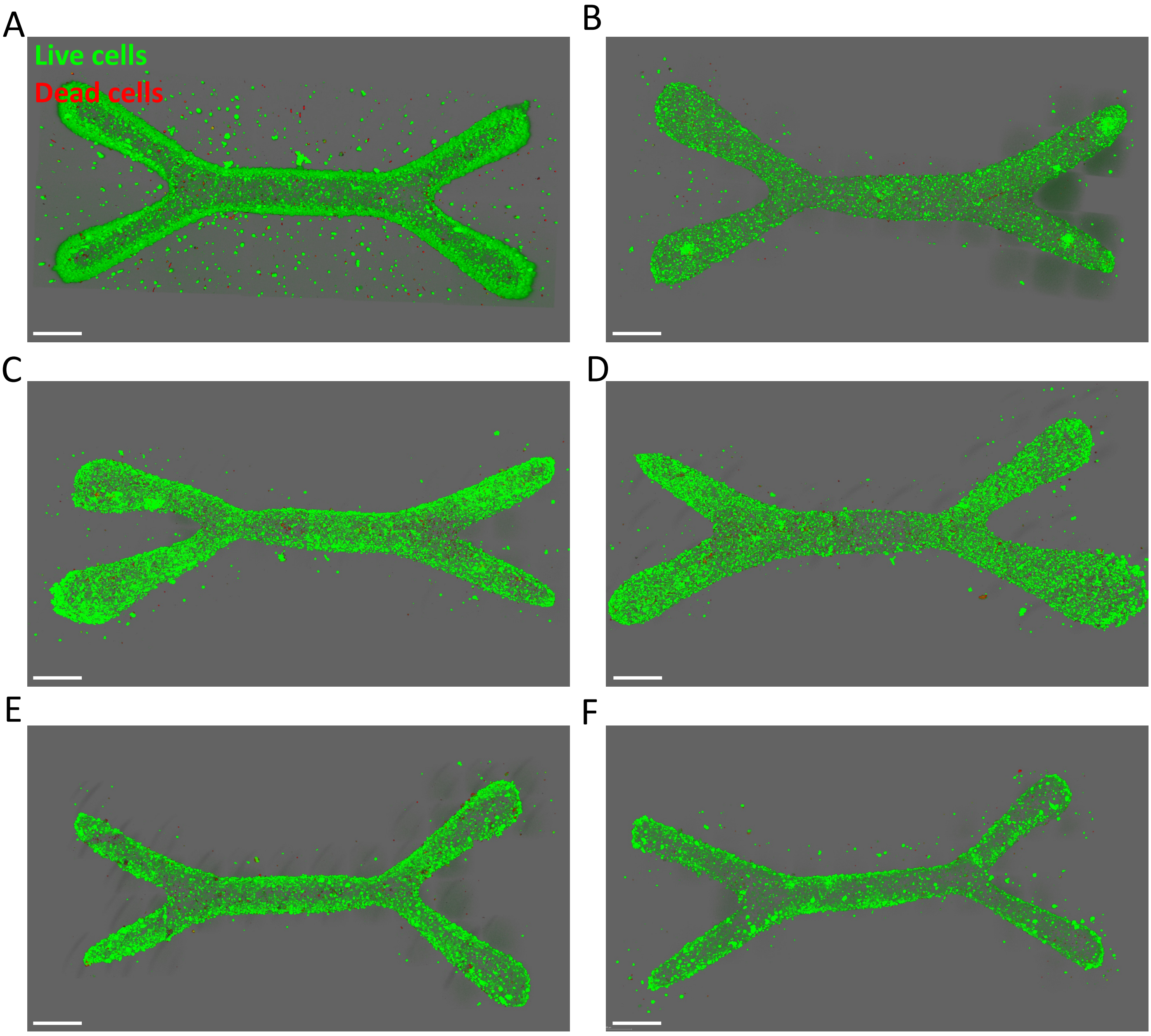

Fig. S2. (A-F) 3D reconstructions from confocal imaging of LIVE/DEAD assays performed on different tissues made from C2C12, just after they were magnetically bioprinted (Live cells in green, dead cells in red, scale bar = 500µm)

A

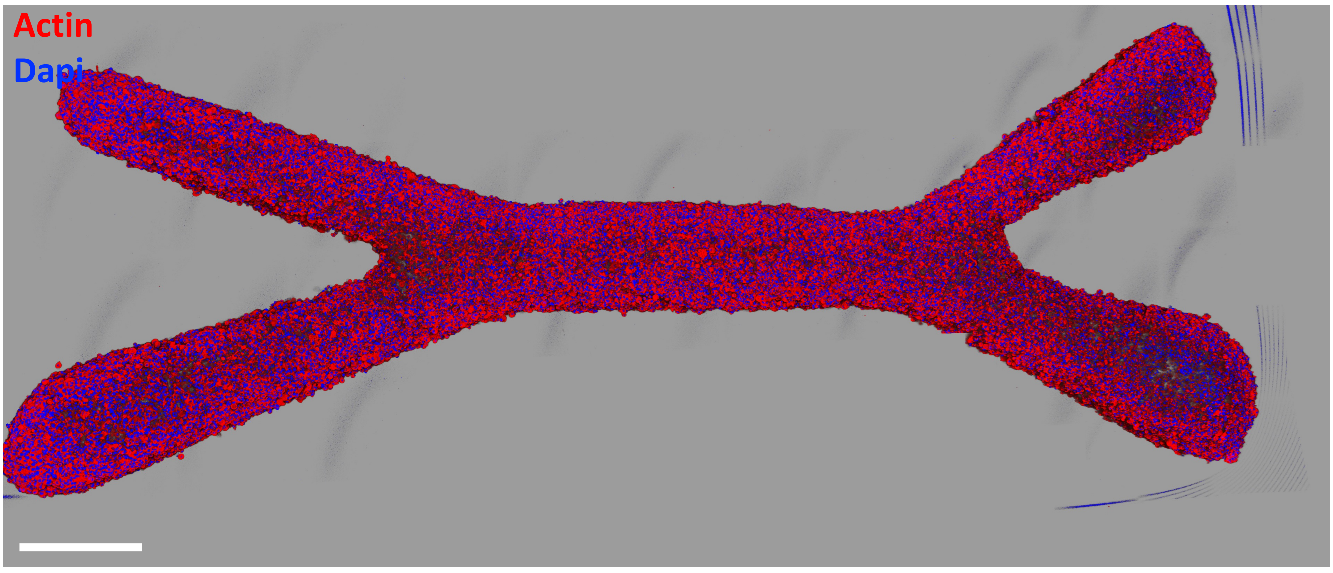

B

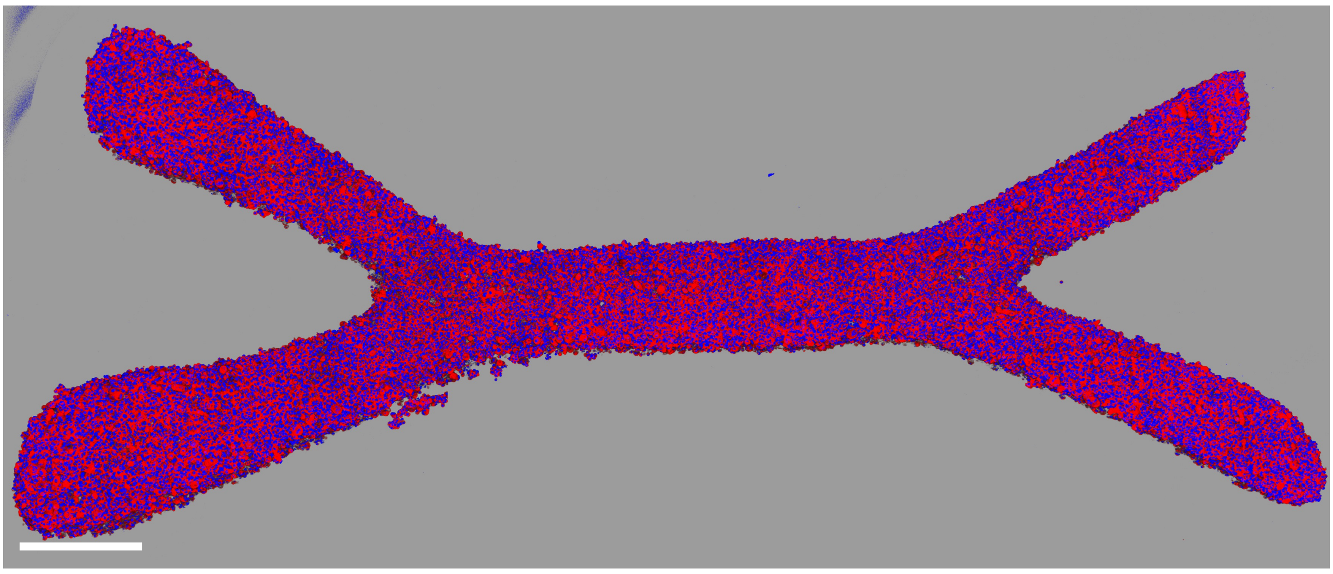

Fig. S3. (A,B) 3D reconstructions from confocal imaging of different tissues made from C2C12 cells, just after they were magnetically bioprinted (Actin in red, nuclei in blue, scale Bar=500µm)

Trapping the magnetic-printed muscle tissue between two magnetic needles

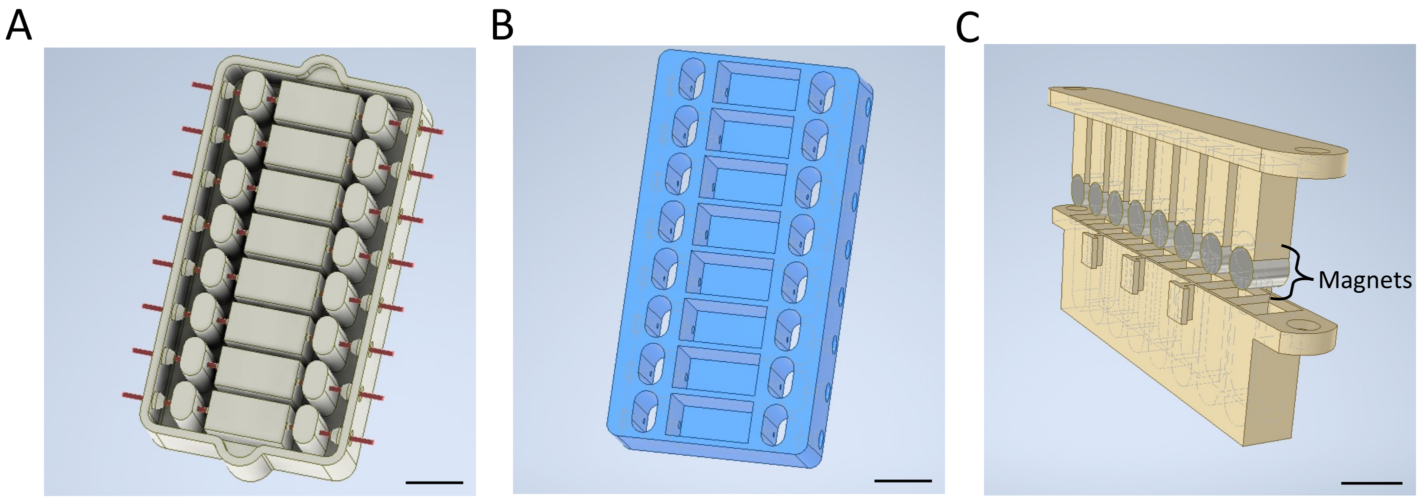

Fig. S4 . Designs of (A) the mold with needles inserted in it, (B) the resulting chip and (C) the opened-up system to hold the eight 6x6mm cylindrical magnets together, made with Autodesk Inventor (Scale bar=1cm)

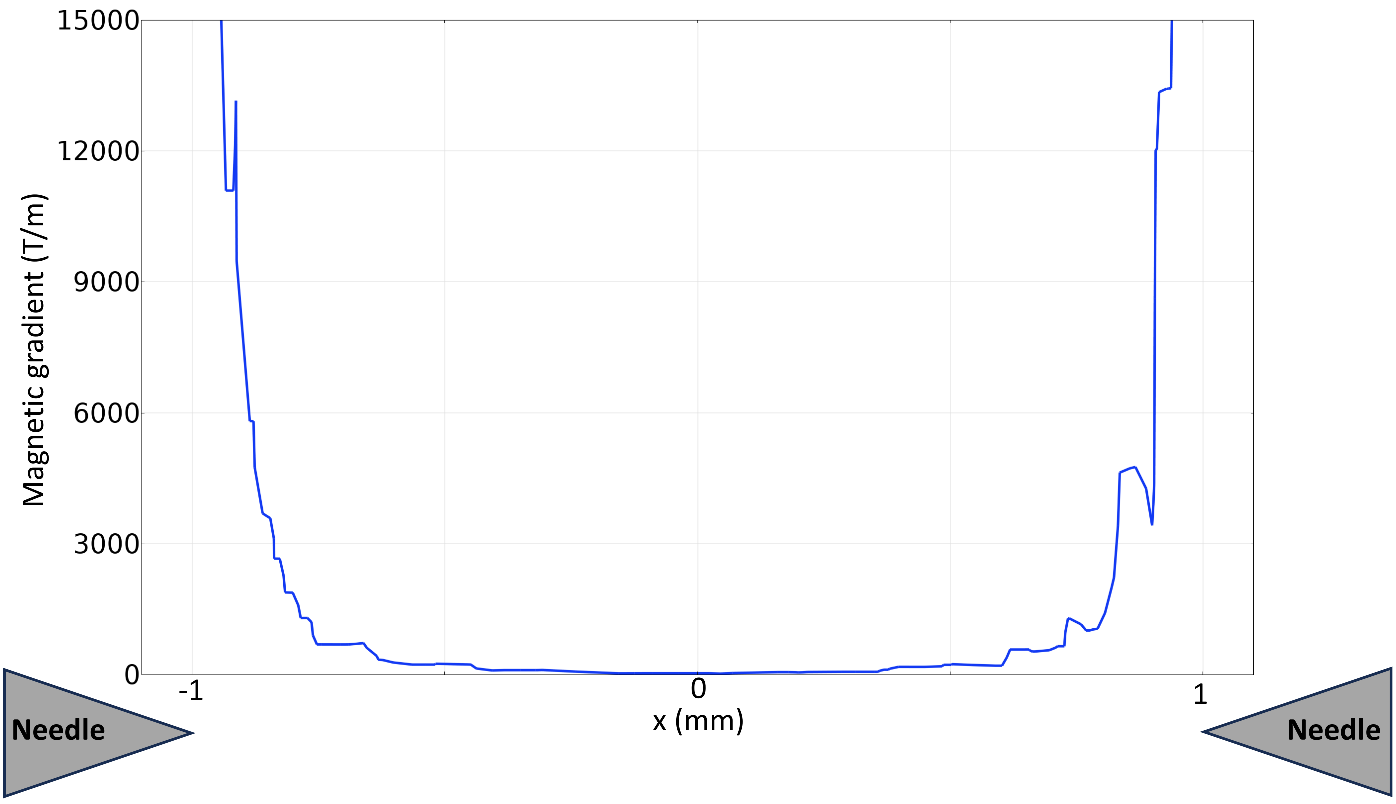

Fig. S5 . Plot of the magnetic gradient between two needles in the chip along their axis, generated from Comsol simulations

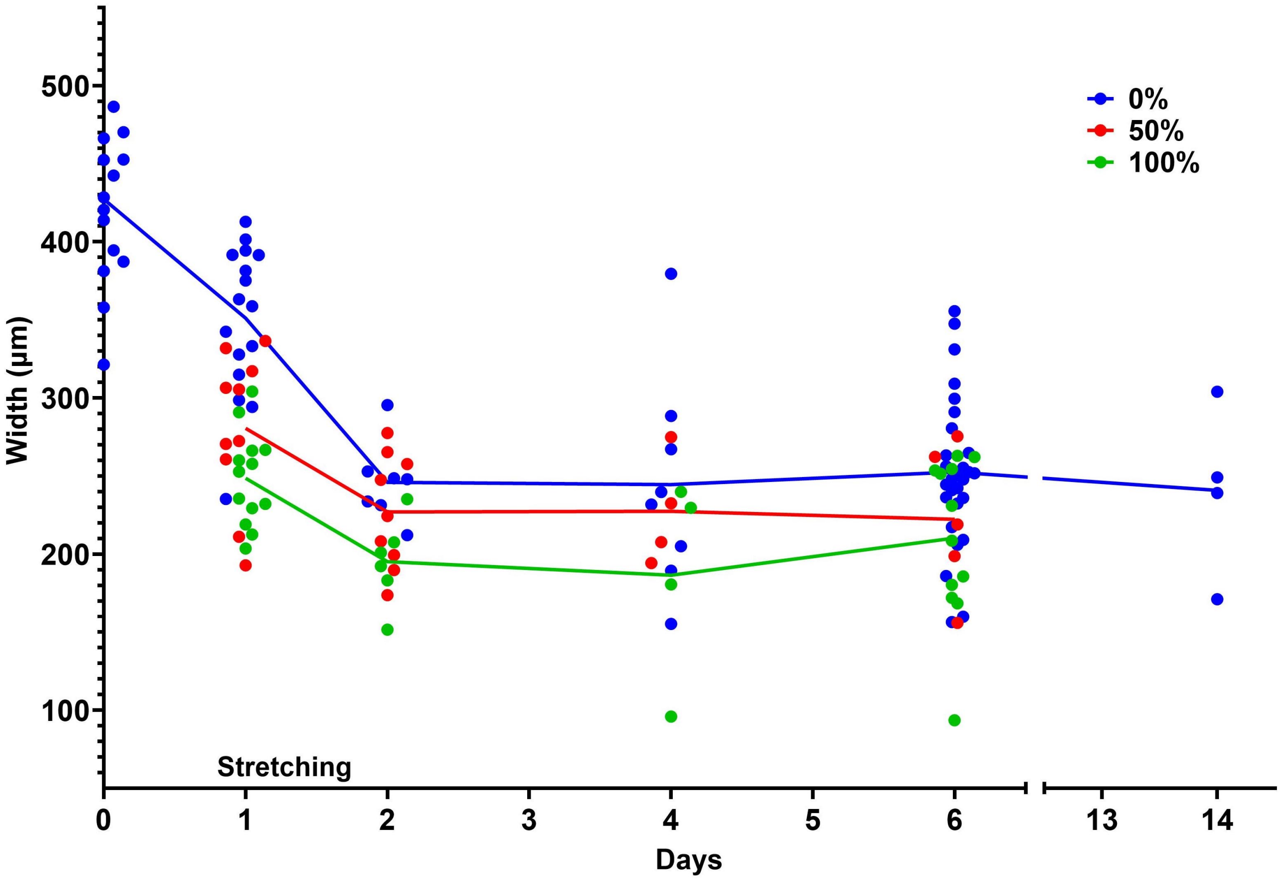

Fig. S6. Evolution of the widths of tissues made of C2C12 cells, whether they were stretched by 0%, 50% or 100% at day 1

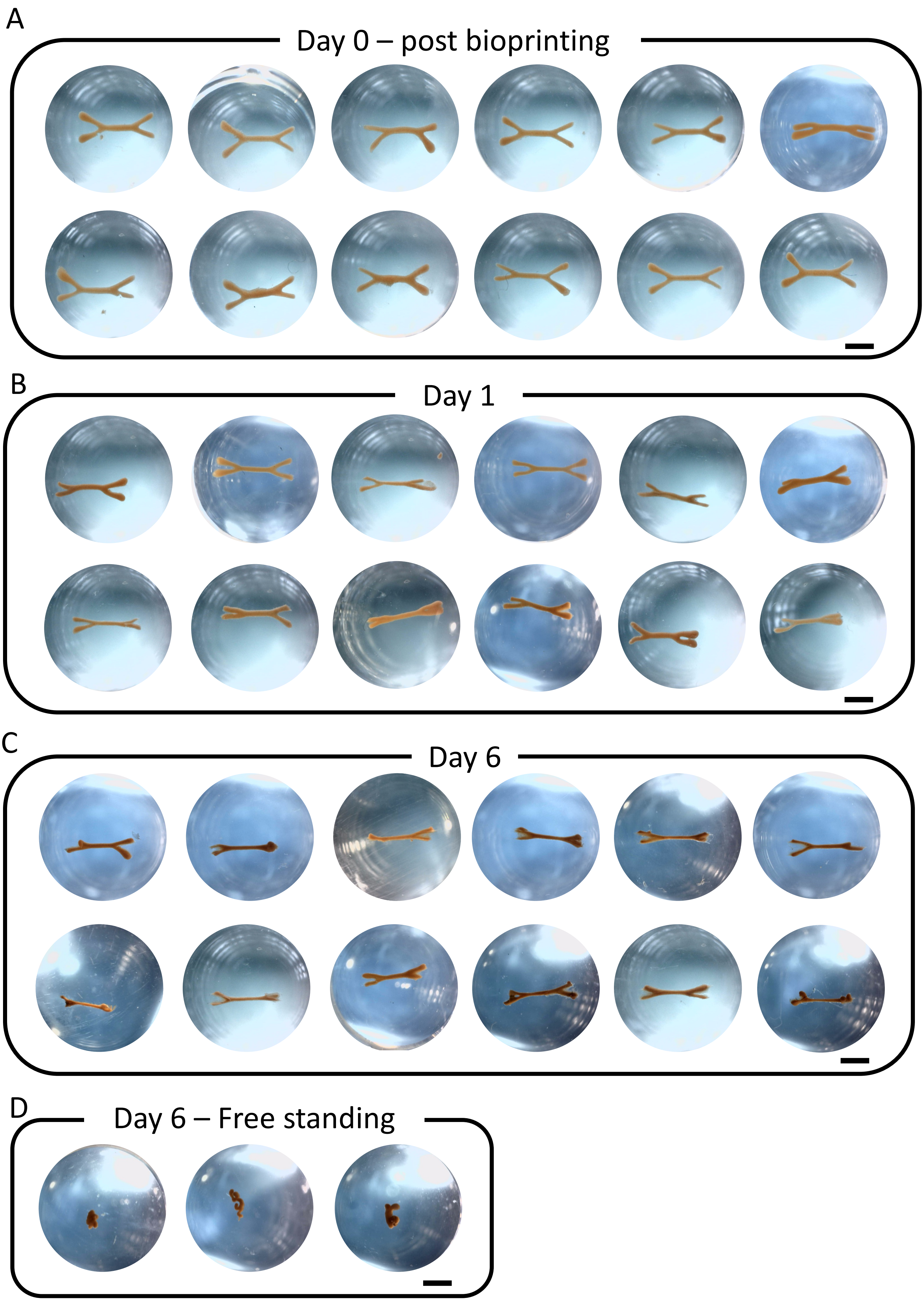

Fig. S7. Different tissues made of C2C12 cells that were fixed and imaged in PBS in the wells of the 48-well plate in which they were preserved. 3D issues that were fixed (A) at day 0 just after being bioprinted, and tissues cultured trapped on the needles fixed (B) at day 1 and (C) at day 6 . (D) Tissues that were cultured free standing in a non adherent well collected at day 6. Tissues that were trapped between needles were fixed still trapped in the chip, then removed from the needles by scrapping the clamps with tweezers and placed in PBS filled wells. They were imaged from the top using a magnifier and a dino camera. (Scale bar= 2mm)

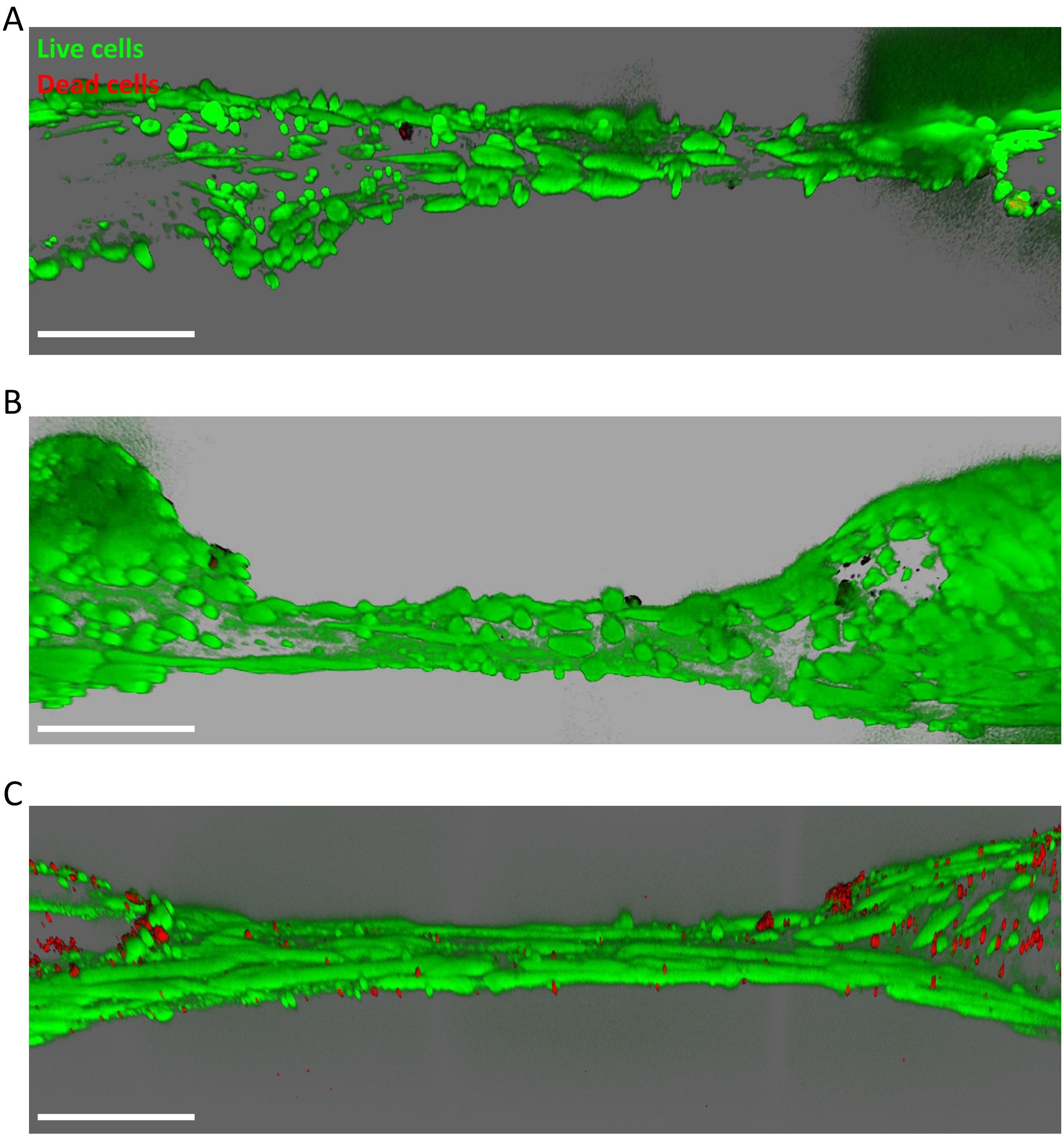

Fig. S8. (A-C) 3D reconstructions from confocal imaging of LIVE/DEAD assays performed on different non stretched 6 day-old tissues made from C2C12 cells, imaged in the chip still trapped on the needles (Live cells in green, dead cells in red, scale bar=500µm)

Preserving tissue anisotropy for myogenesis

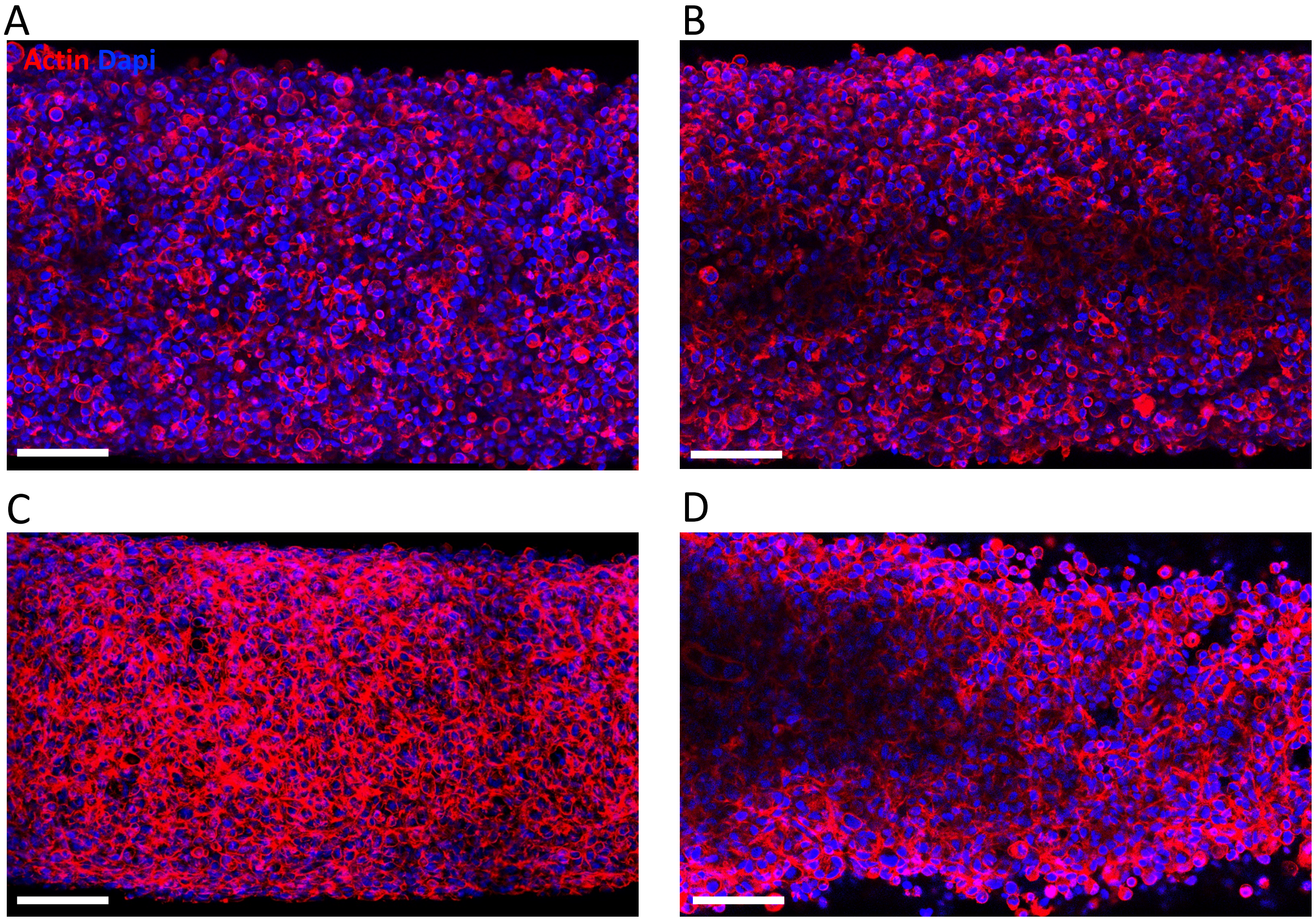

Fig. S9. Confocal imaging of different tissues made of C2C12 cells at day 0, just after bioprinting (Actin in red, nuclei in blue, scale bar = 100µm)

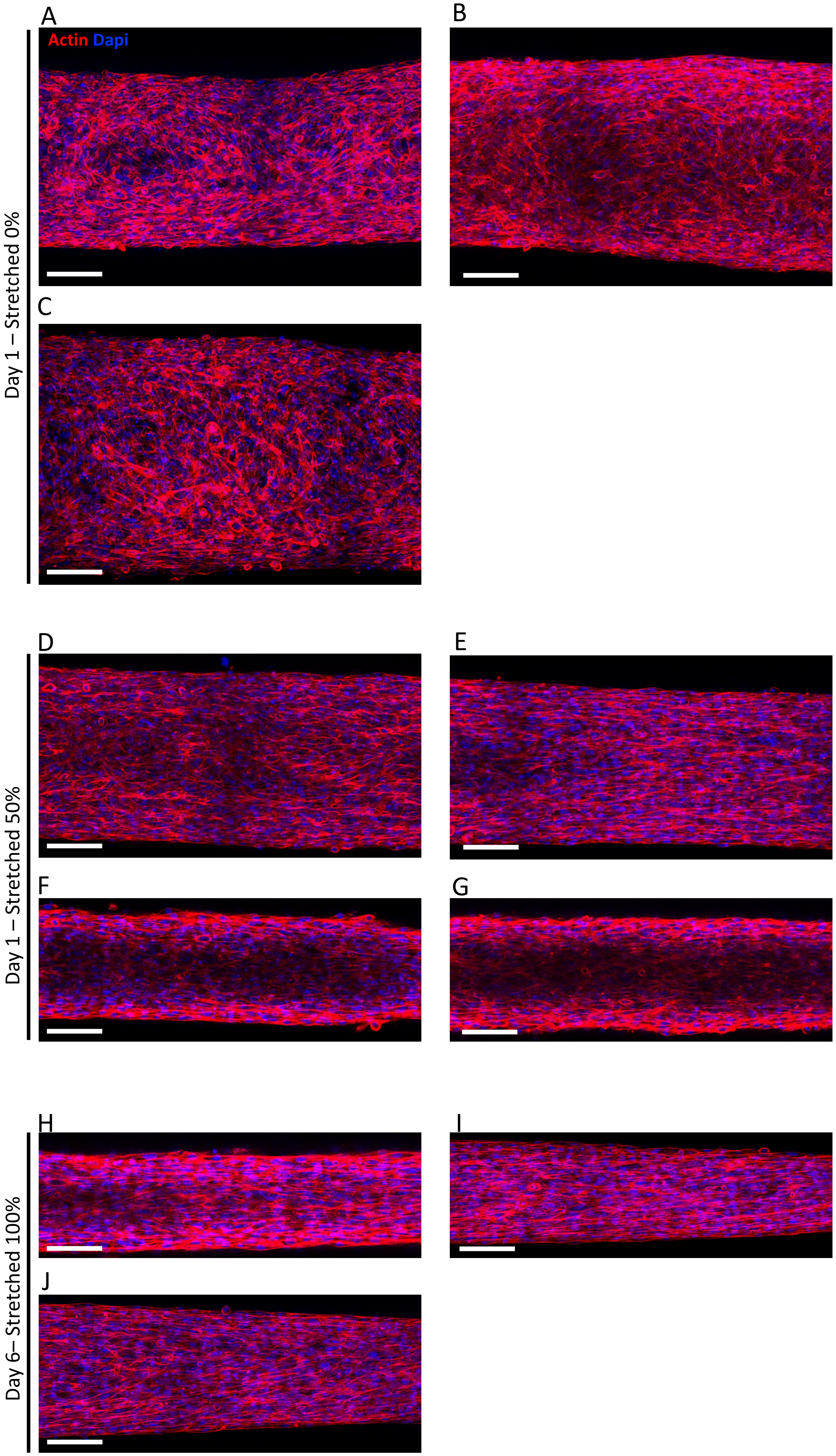

Fig. S10. Confocal imaging of different tissues made of C2C12 cells at day 1, which were stretched by (A-C) 0%, (D-G) 50% and (H-J) 100% (Actin in red, nuclei in blue, scale bar=100µm)

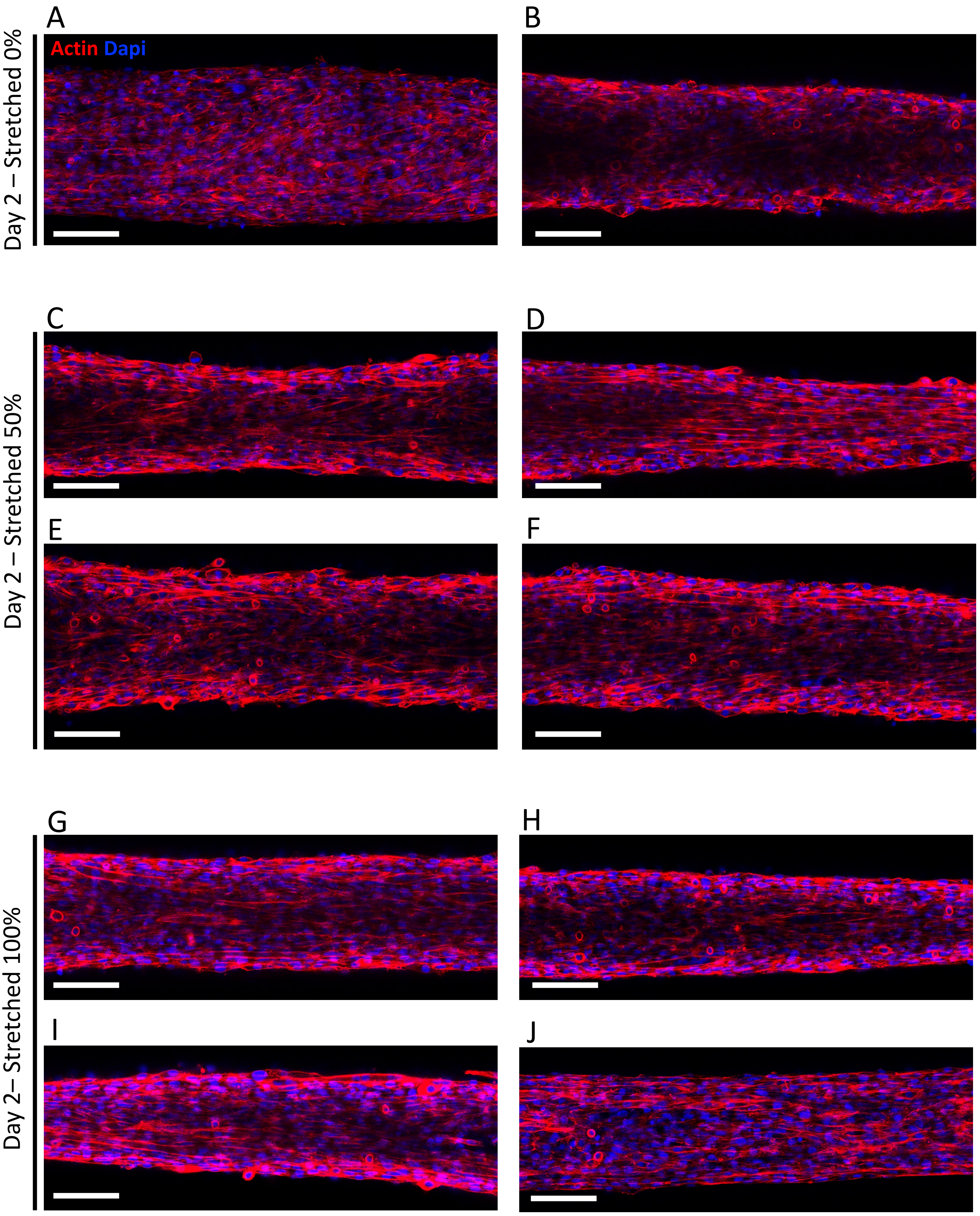

Fig. S11. Confocal imaging of different tissues made of C2C12 cells at day 2, which were stretched since day 1 by (A-B) 0%, (C-F) 50% and (G-J) 100% (Actin in red, nuclei in blue, scale bar = 100µm)

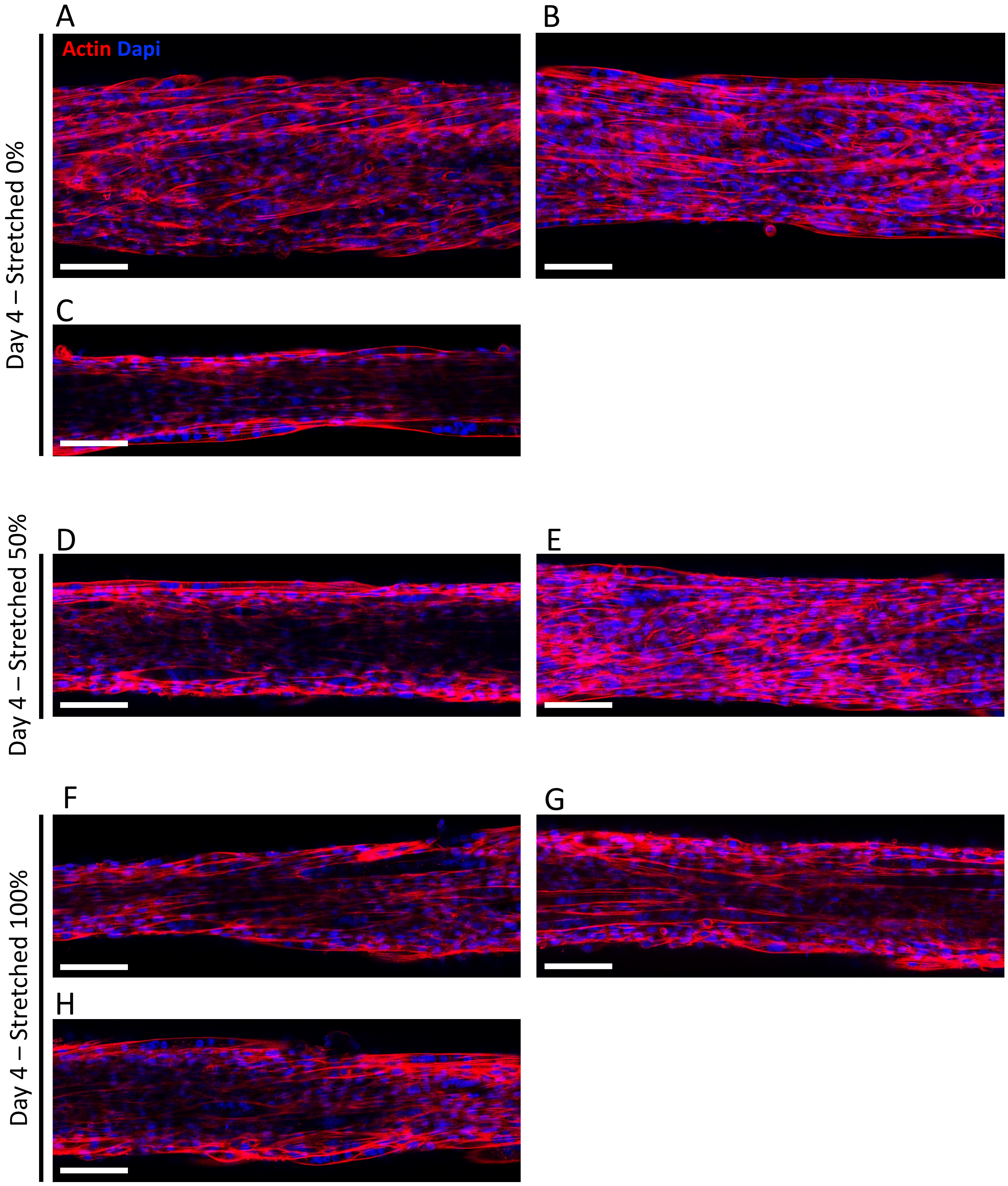

Fig. S12. Confocal imaging of different tissues made of C2C12 cells at day 4, which were stretched since day 1 by (A-C) 0%, (D-E) 50% and (F-H) 100% (Actin in red, nuclei in blue, scale bar = 100µm)

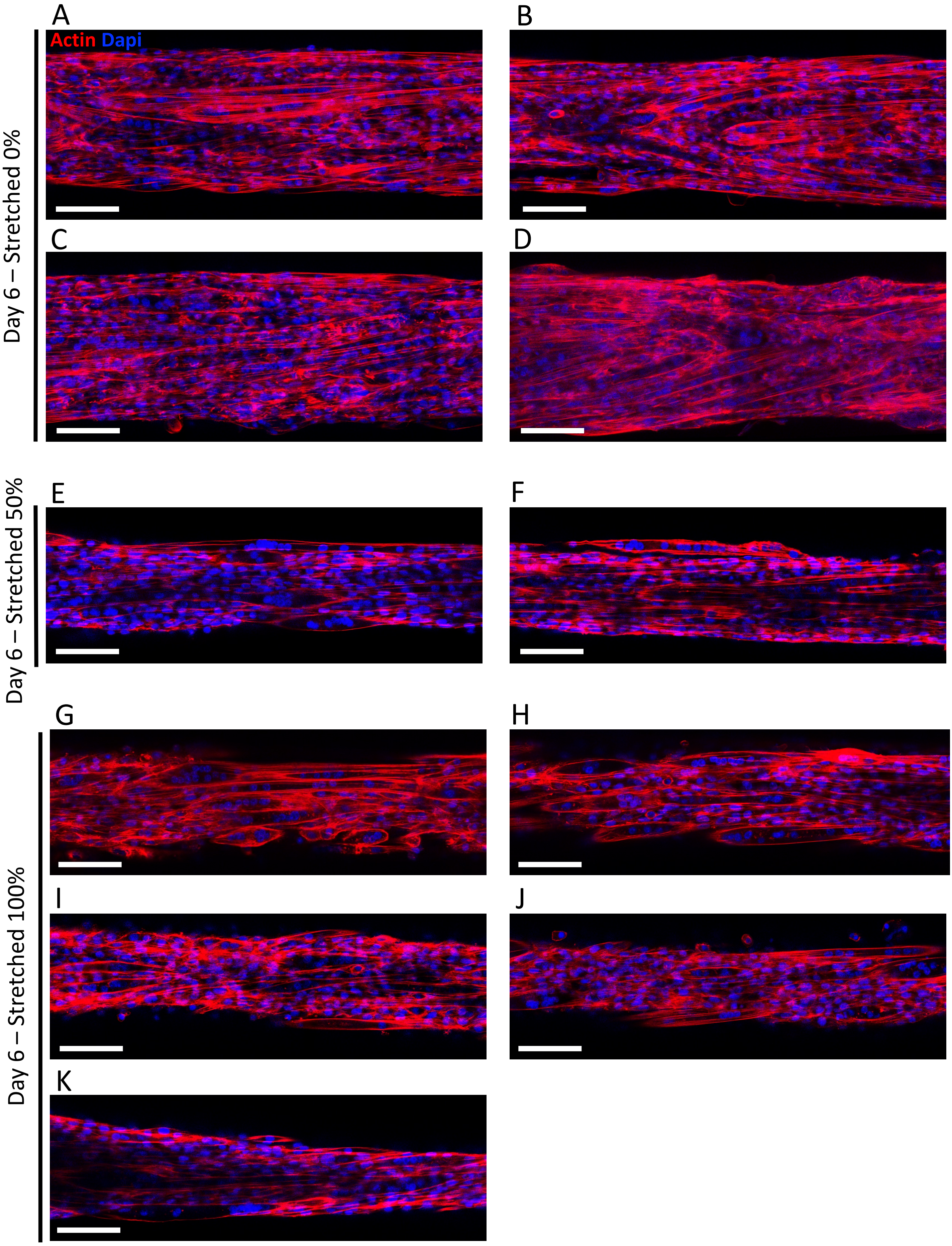

Fig. S13. Confocal imaging of different tissues made of C2C12 cells at day 6, which were stretched since day 1 by (A-D) 0%, (E-F) 50% and (G-K) 100% (Actin in red, nuclei in blue, scale bar=100µm)

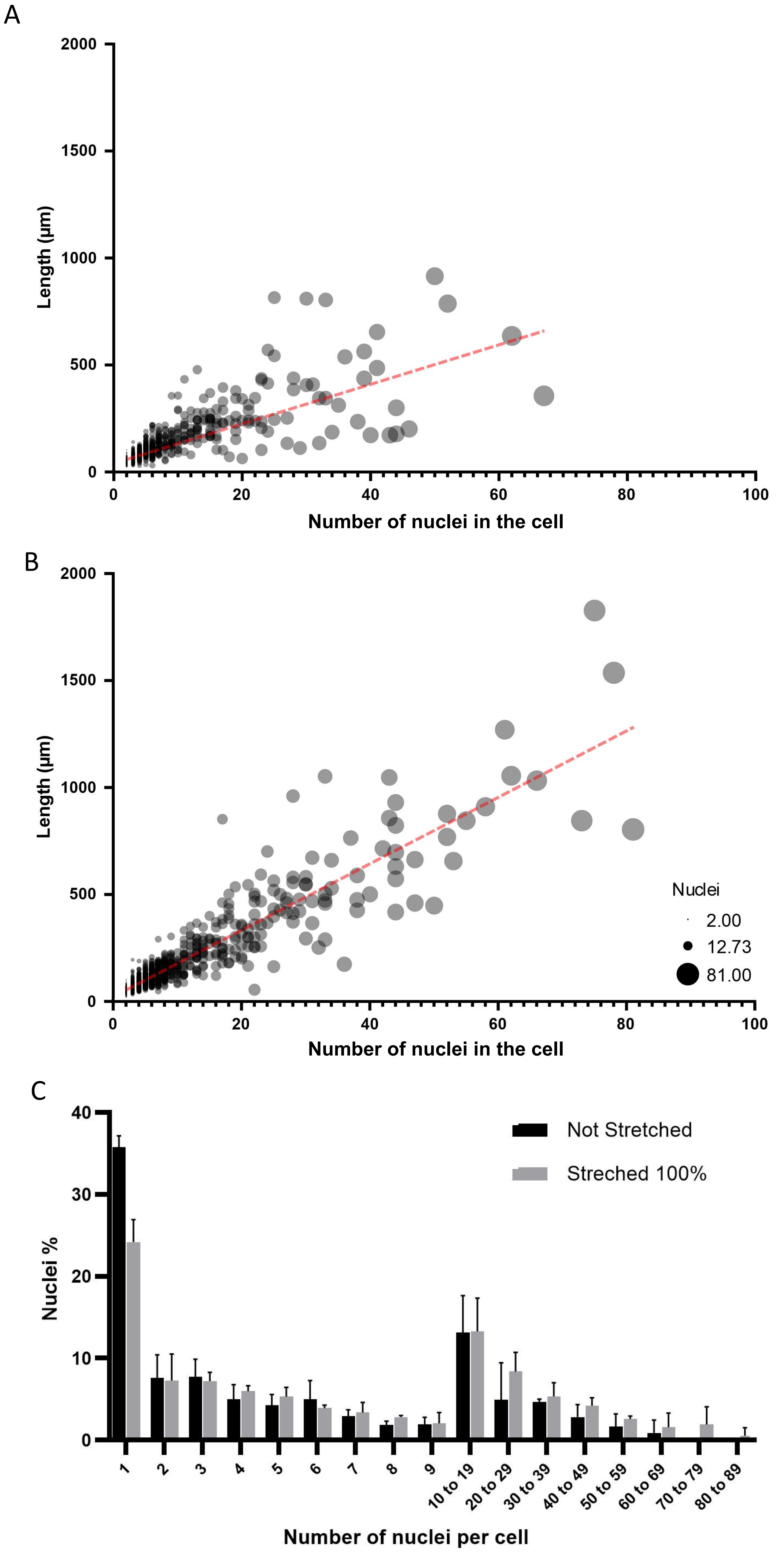

Fig. S14 . (A-B) Scatter plots of the lengths of mulitnucleated cells as a function of the number of nuclei in the cells for 6 day old tissues that were stretched by (A) 0% (n=3) and (B) 100% since day 1 (n=3). The diameter of each point is proportionate to the number of nuclei in the cell. A trendline in red was plotted from a linear regression. (C) Distribution of nuclei fusion in terms of nuclei percentage at day 6 in 3D tissues that were either not stretched or stretched by 100%.

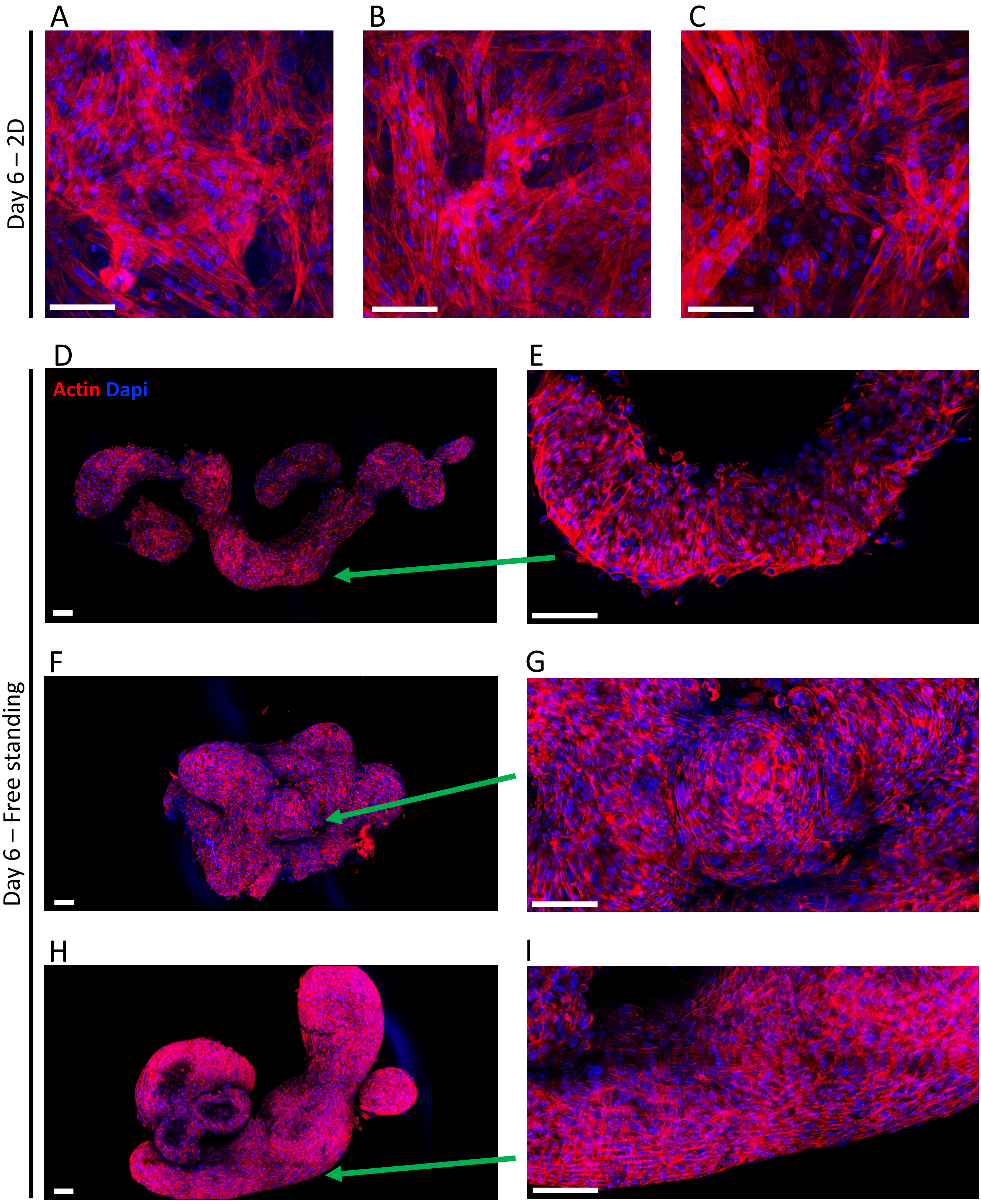

Fig. S15. Confocal imaging at day 6 of (A-C) C2C12 cells cultured in 2D and of (D-I) different 3D tissues which were not trapped between needles and instead were left free standing in non-adherent wells (Actin in red, nuclei in blue, scale bar = 100µm)

Stretching improves muscle differentiation

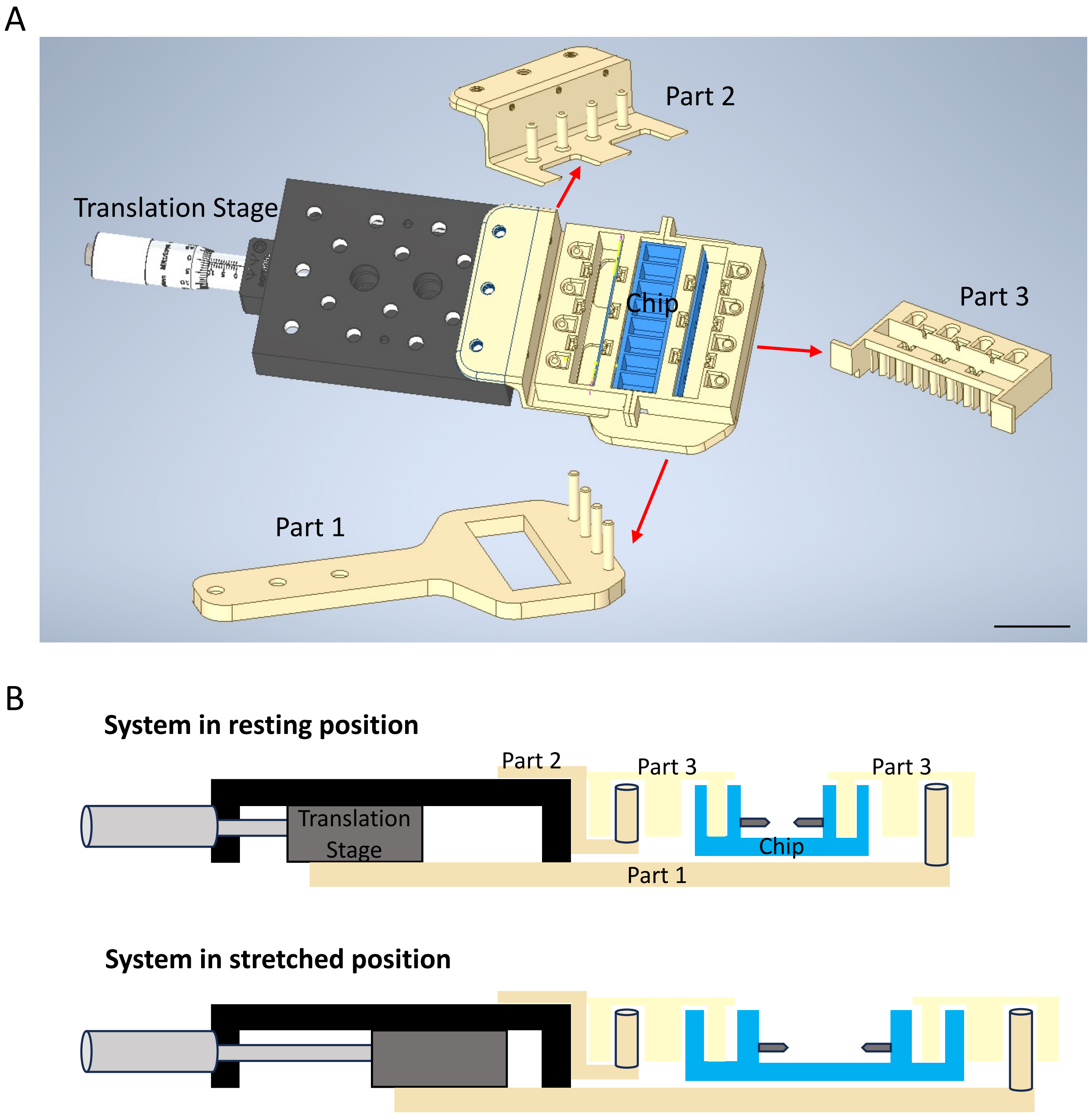

Fig. S16 . (A) Designs of the system stretching the chip and the individual 3D printed parts composing it, made with Autodesk Inventor (Scale bar=3cm), and (B) simplified shematics showing how these different parts are organized from a longitudinal cut when the system is in its resting position or stretched position.

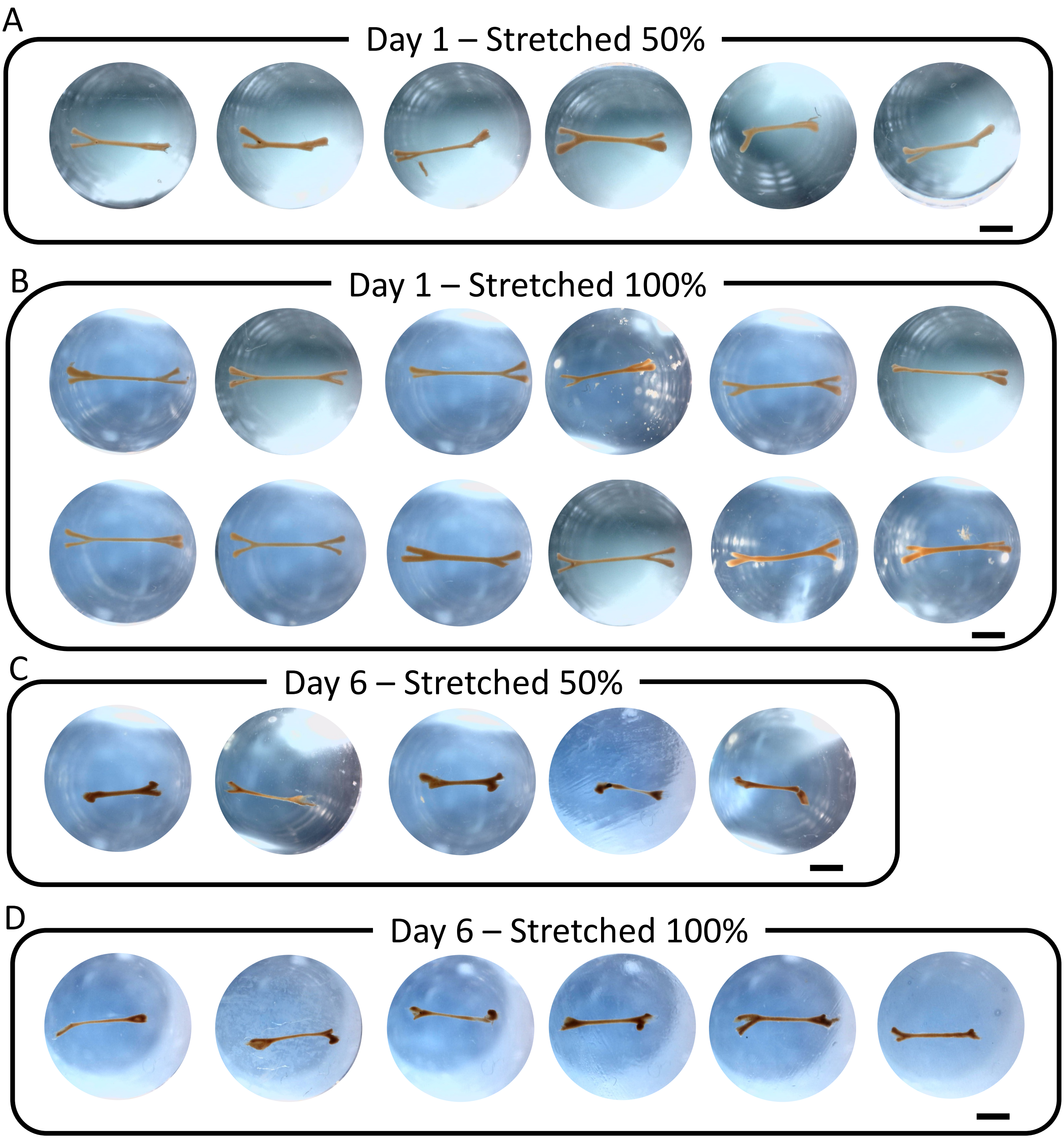

Fig. S17. Different tissues made of C2C12 cells that were fixed and imaged in PBS in the wells of the 48-well plate in which they were preserved. Tissues collected on day 1 just after they were stretched by (A) 50% or (B) 100% and on day 6 which were stretched since day 1 by (C) 50% or (D) 100%. Tissues that were trapped between needles or stretched were fixed still trapped or stretched in the chip, then removed from the needles by scrapping the clamps with tweezers and placed in PBS filled wells. They were imaged from the top using a magnifier and a dino camera. (Scale bar = 2mm)

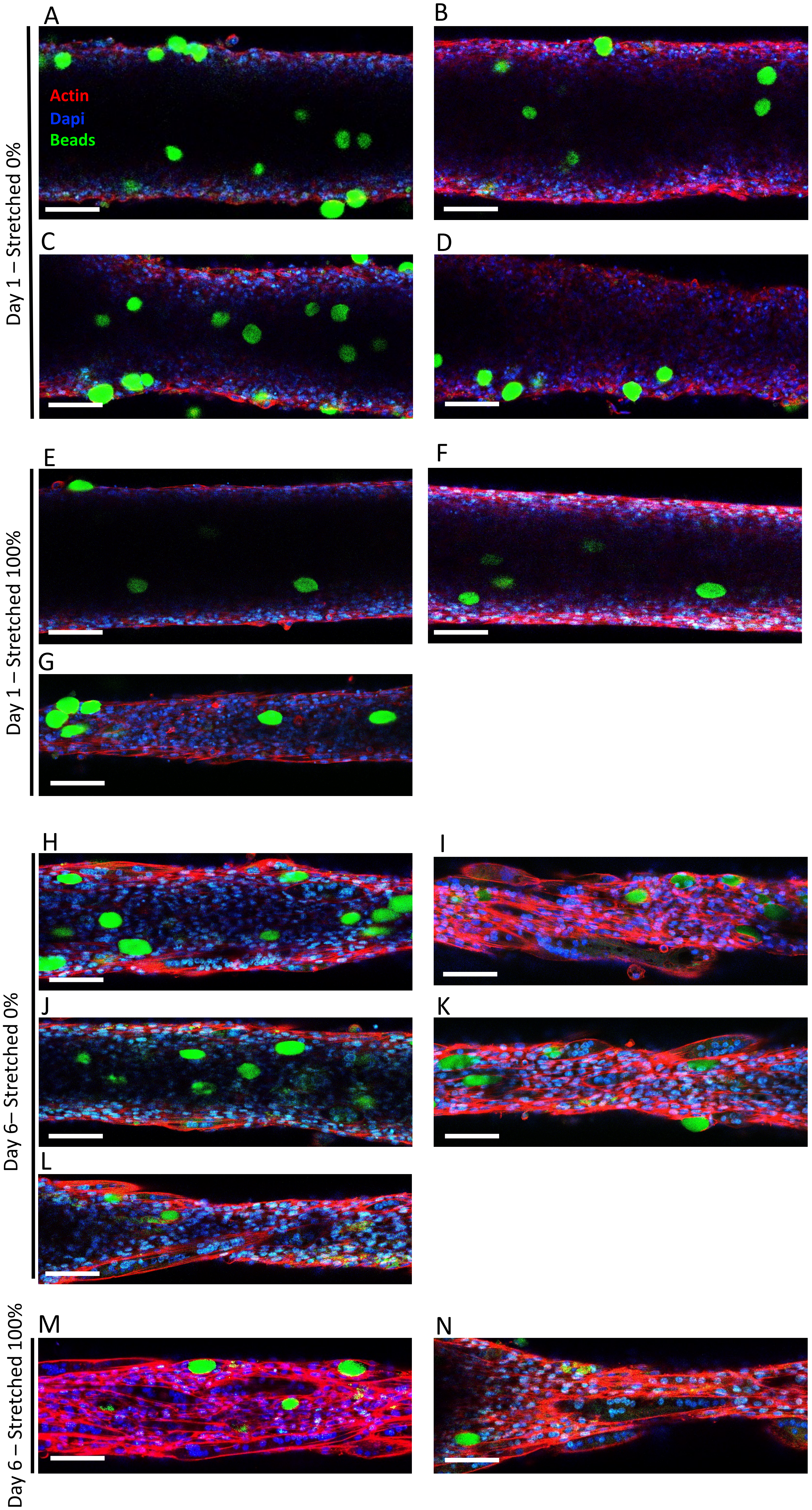

Fig. S18. Confocal imaging of different tissues made of C2C12 cells, in which polyacrylamide beads were embedded, at day 1 after they were stretched by (A-D) 0% or (E-G) 100%, and at day 6 which were stretched since day 1 by (H-L) 0% or (M-N) 100%. (Actin in red, dapi in blue, beads in green, scale bar = 100 µm)

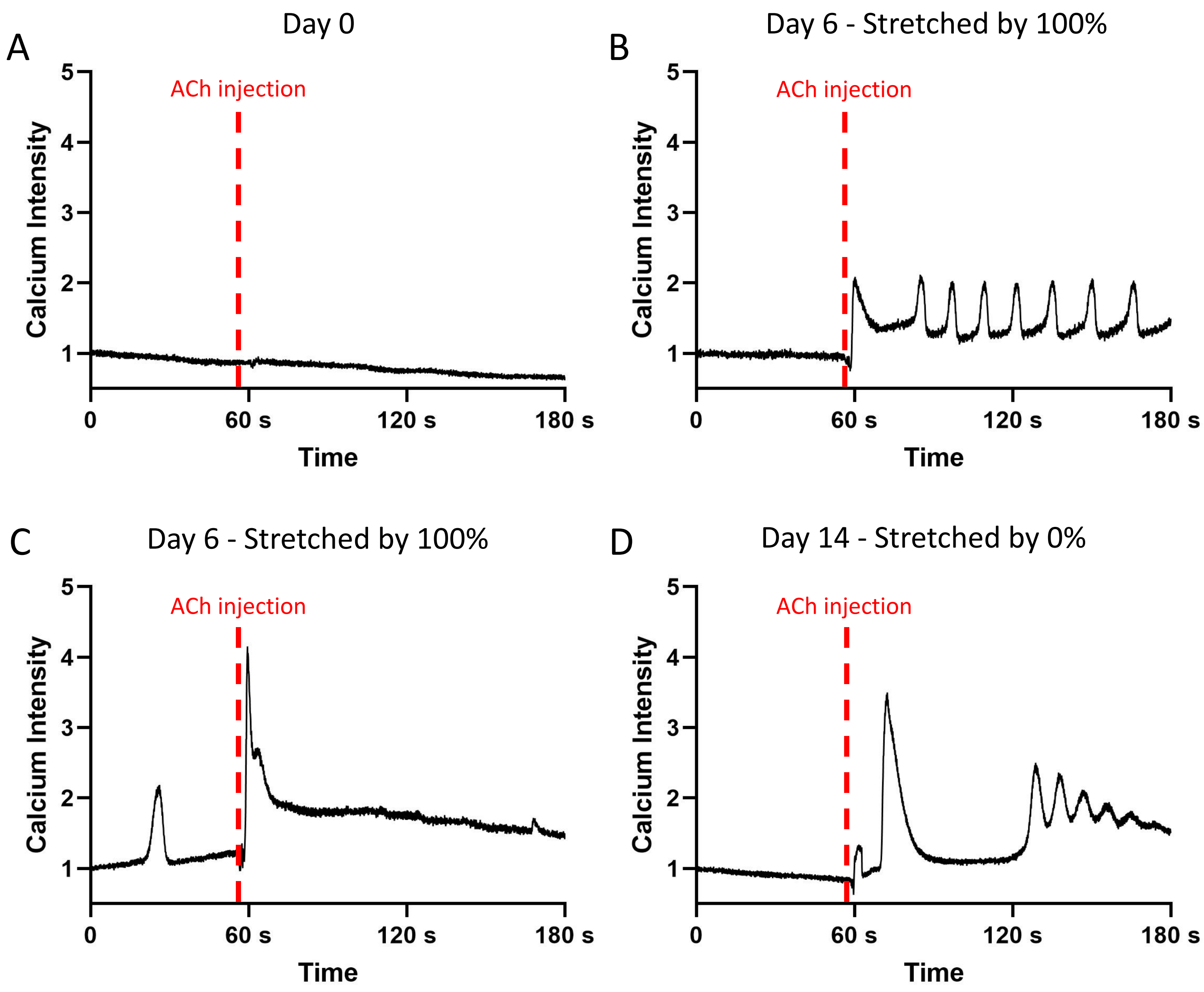

Fig. S19. Kymographs of the fluctuations of the calcium signal in (A) a C2C12 cell from a tissue at day 0 just after bioprinting, (B-C) in two different C2C12 cells from tissues at day 6 stretched by 100% since day 1, and (D) a C2C12 cell from a non-stretched tissue at day 14, treated with 1mM ACh after approximately 1min.

Transposing to myoblasts derived from human induced pluripotent stem cells

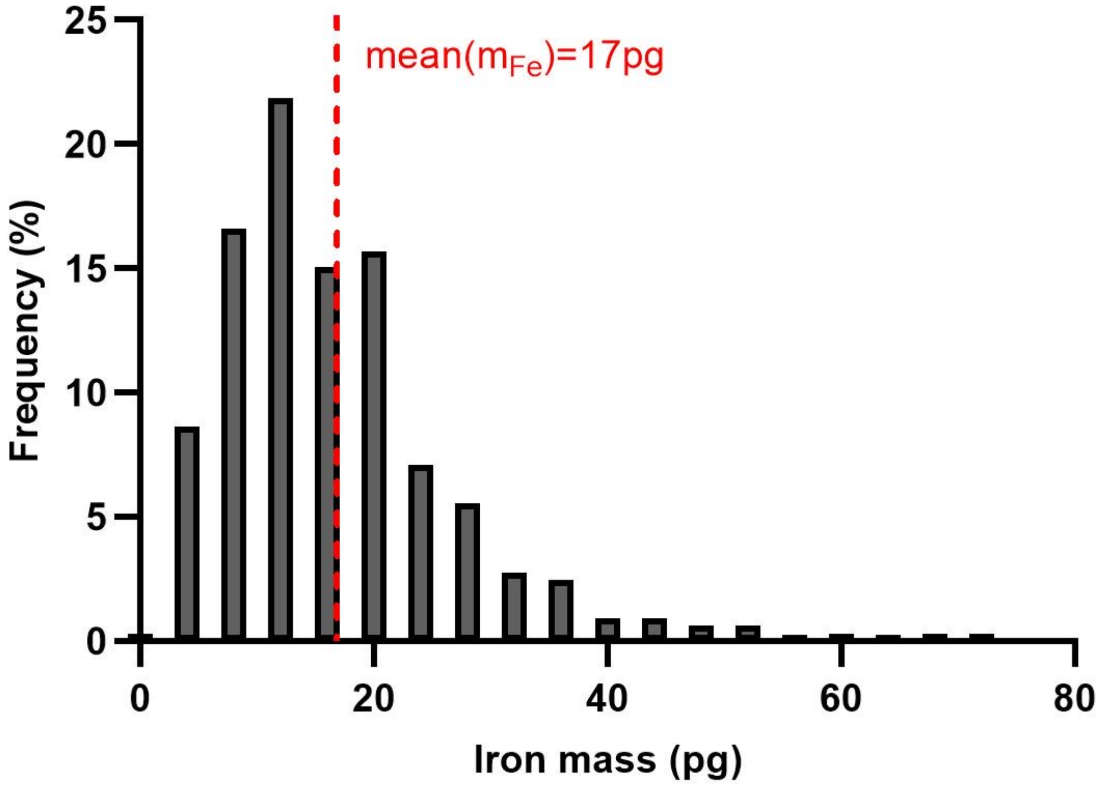

Fig. S20 . Histogram of the distribution of iron mass internalized per cell for M-ihPSC 1 collected just before patterning, after they were labeled with 2mM of iron and 2mM citrate in RPMI for 15mn once a day for the two previous days and with 0.2mM of iron in their proliferation medium overnight between these two days

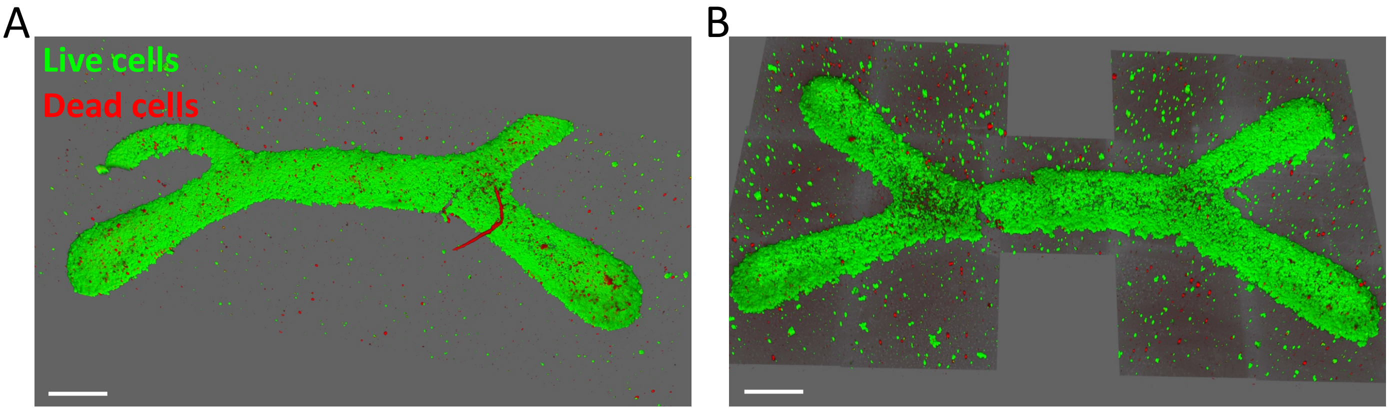

Fig. S21 . 3D reconstructions from confocal imaging of LIVE/DEAD assays performed on different tissues made from M-hiPSC 1, just after they were magnetically bioprinted (Live cells in green, dead cells in red, scale bar=500µm)

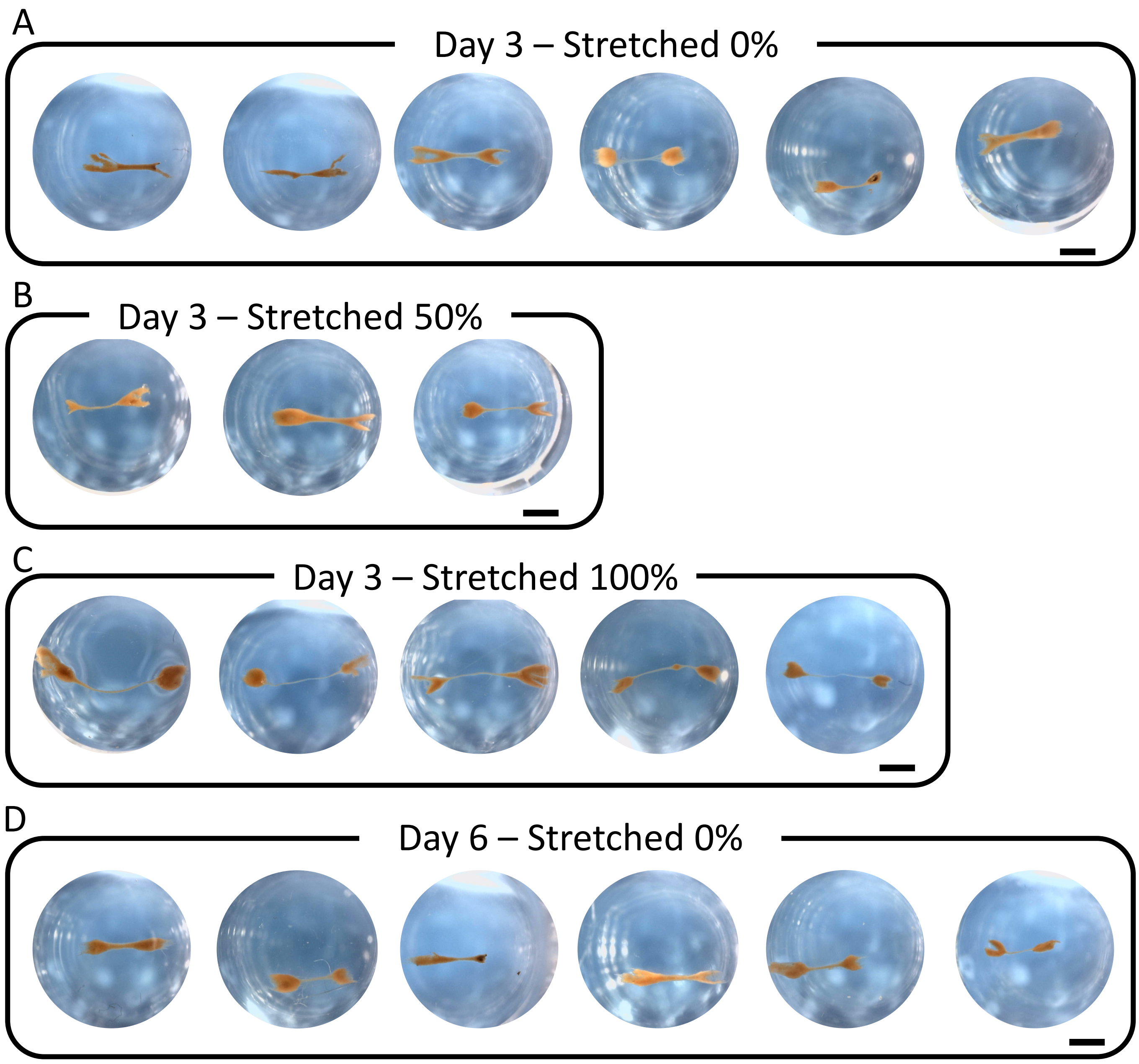

Fig. S22. Different tissues made of M-hiPSC 1 that were fixed and imaged in PBS in the wells of the 48-well plate in which they were preserved. Tissues that were fixed at day 3 that were stretched since day 1 by (A) 0%, (B) 50% or (C) 100% and at (D) day 6 without stretching. Tissues that were trapped between needles or stretched were fixed still trapped or stretched in the chip, then removed from the needles by scrapping the clamps with tweezers and placed in PBS filled wells. They were imaged from the top using a magnifier and a dino camera. (Scale bar=2mm)

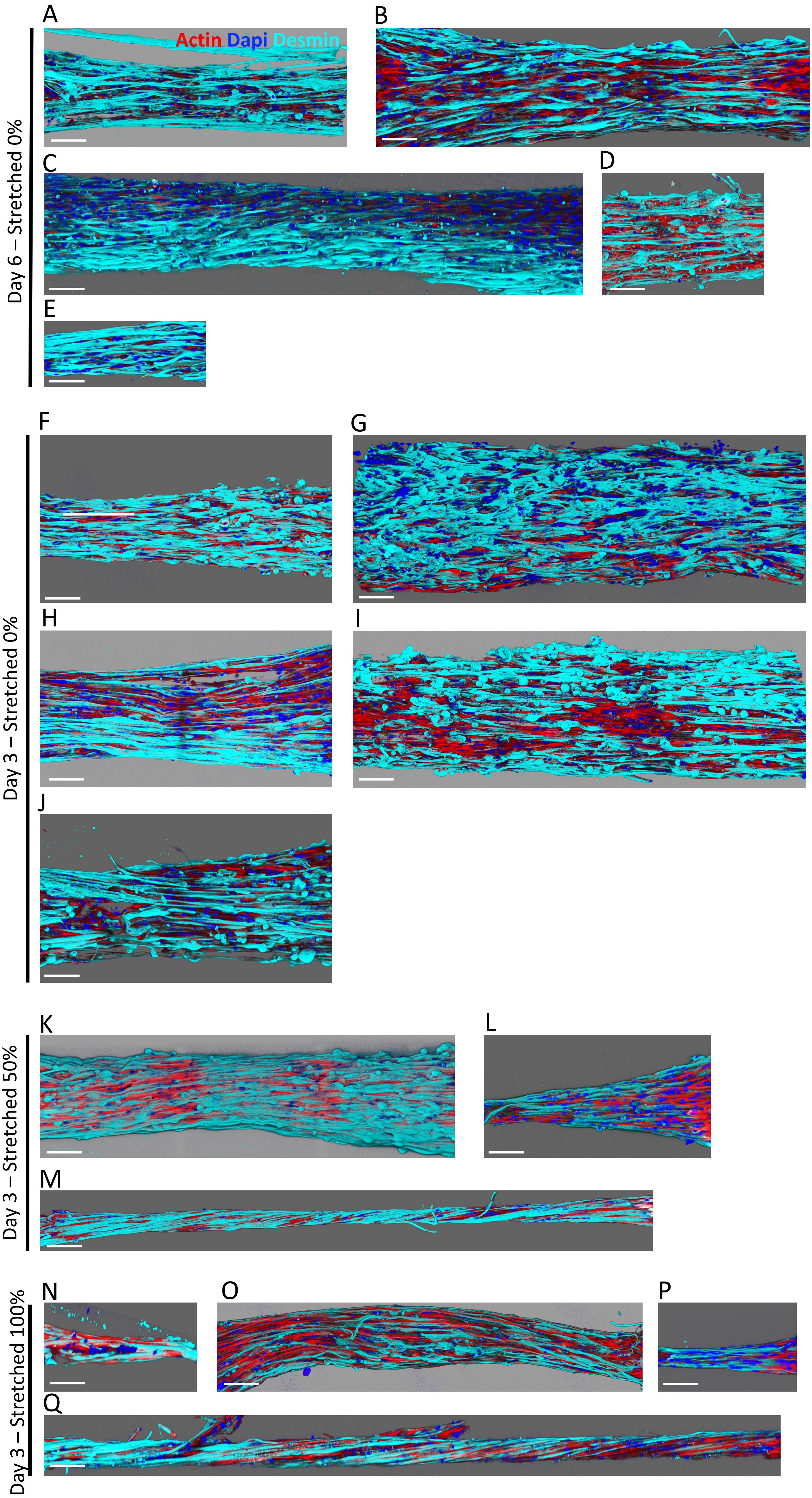

Fig. S23 . 3D reconstruction from confocal imaging of different desmin immunostained tissues made from M-hiPSC 1 cultured for (A-E) 6 days with 0% stretching and 3 days with (F-J) 0%, (K-M) 50% and (N-Q) 100% stretching (actin in red, nuclei in blue, desmin in cyan, scale bar=100µm)

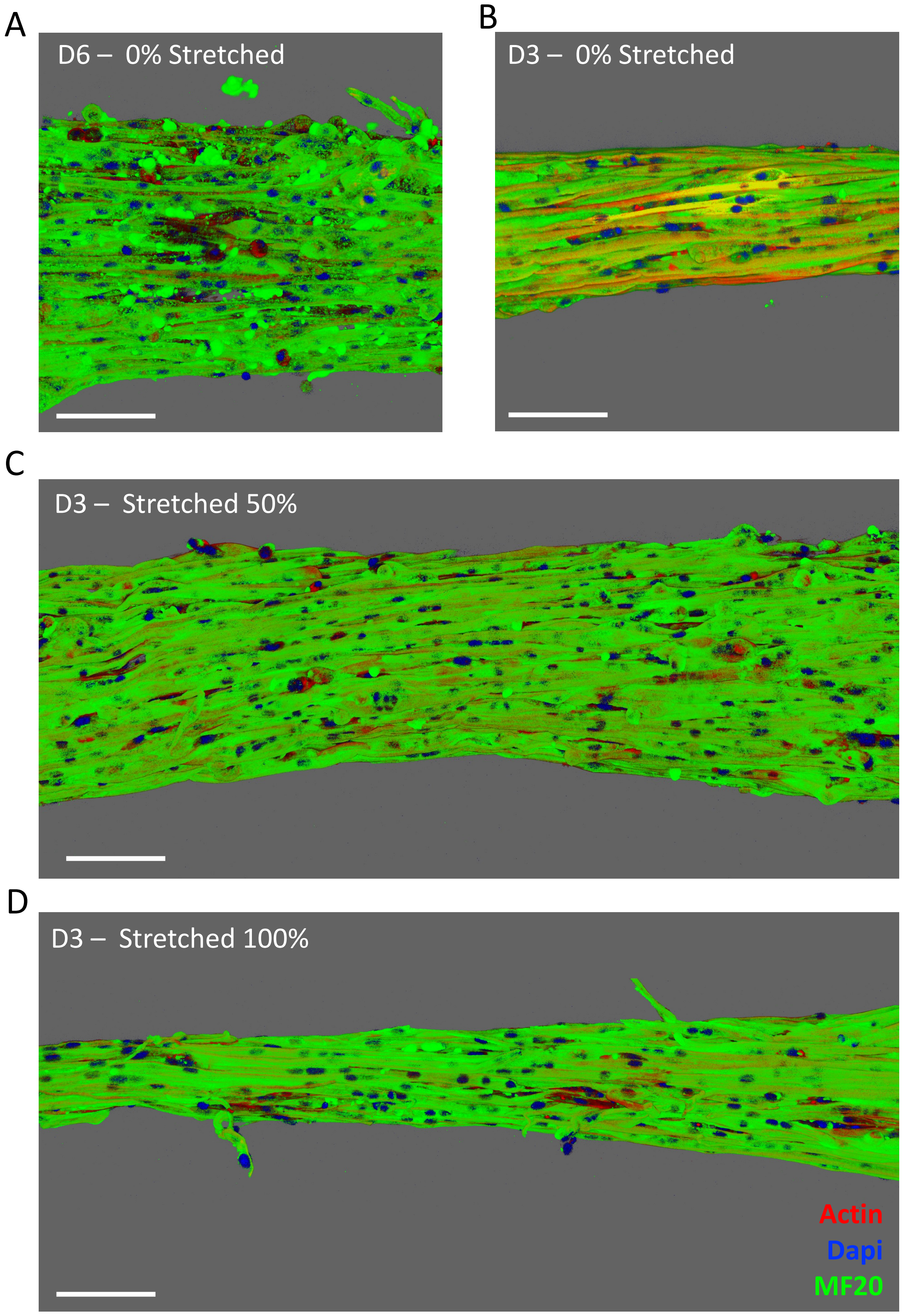

Fig. S24 . 3D reconstruction from confocal imaging of different MF20 immunostained tissues made from M-hiPSC 1 cultured for (A) 6 days with 0% stretching, and 3 days with (B) 0%, (C) 50% and (D) 100% stretching. They were immunostained with MF20 antibody to see myosin heavy chains. (actin in red, nuclei in blue, MF20 in green, scale bar in 100µm)

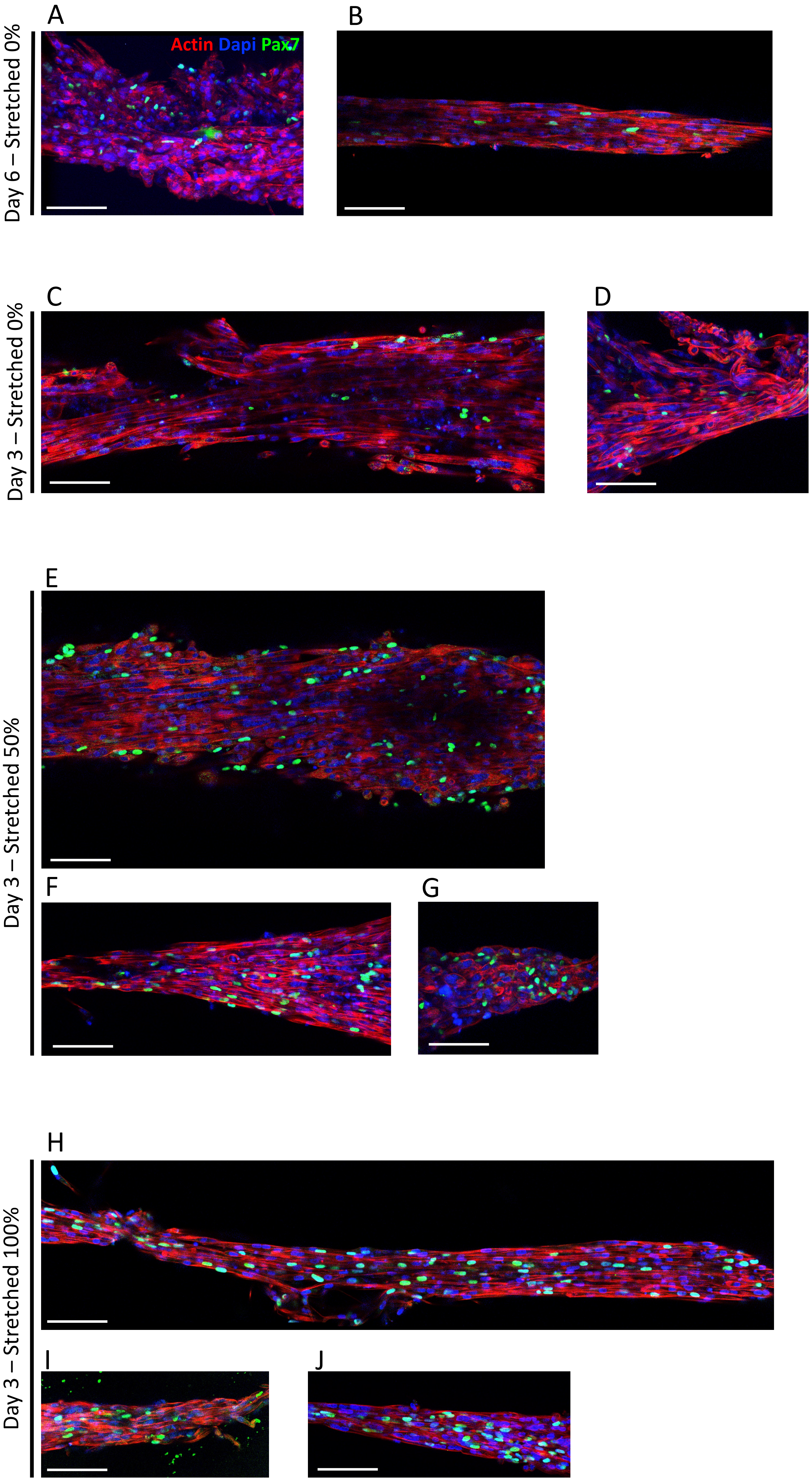

Fig. S25 . Confocal imaging of different Pax 7 immunostained tissues made from M-hiPSC 1 cultured for (A-B) 6 days with 0% stretching and 3 days with (C-D) 0%, (E-G) 50% and (H-J) 100% stretching. (actin in red, nuclei in blue, Pax7 in green, scale bar=100µm)

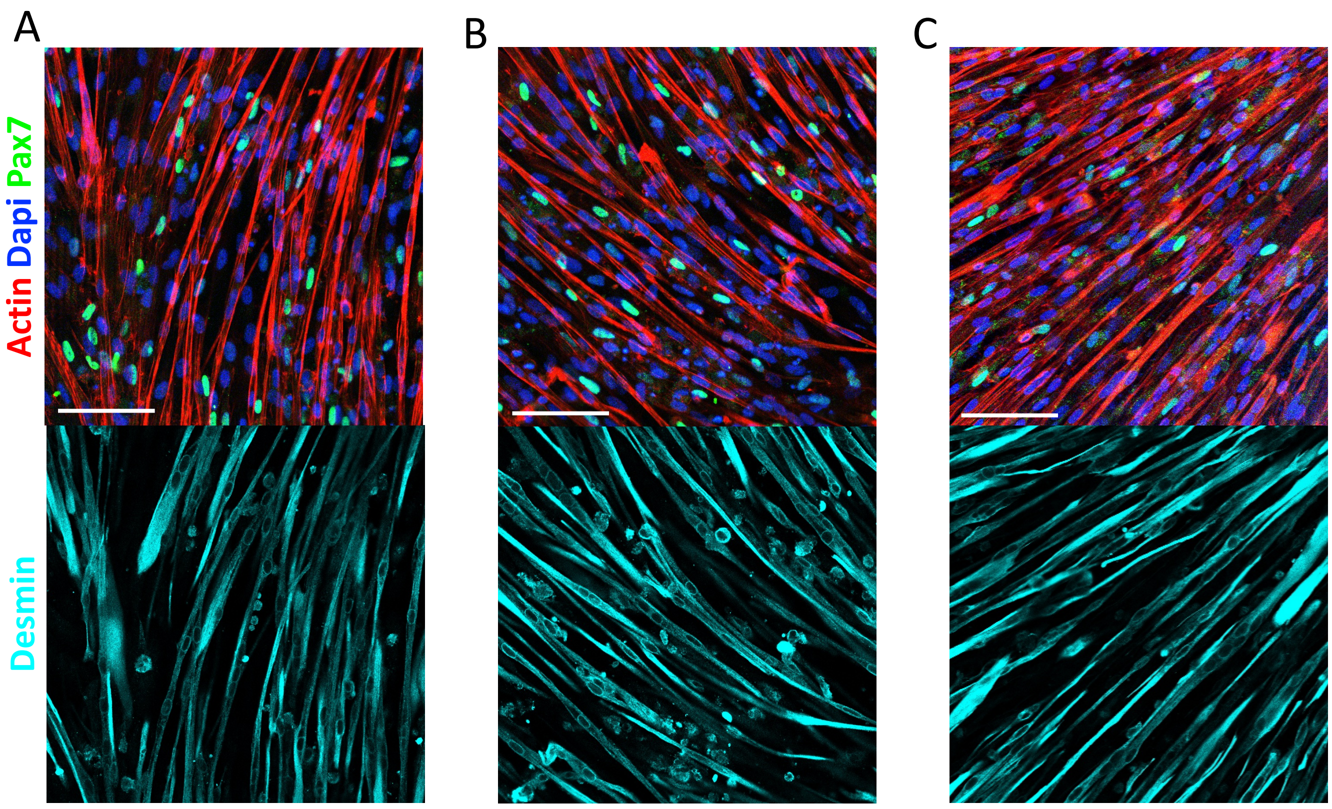

Fig. S26 . Confocal imaging of three different samples of M-hiPSC 1 cultured for 6 days of differentiation in 2D, immunostained for Pax 7 and desmin (Actin in red, nuclei in blue, Pax7 in green, desmin in cyan, scale bar = 100µm)

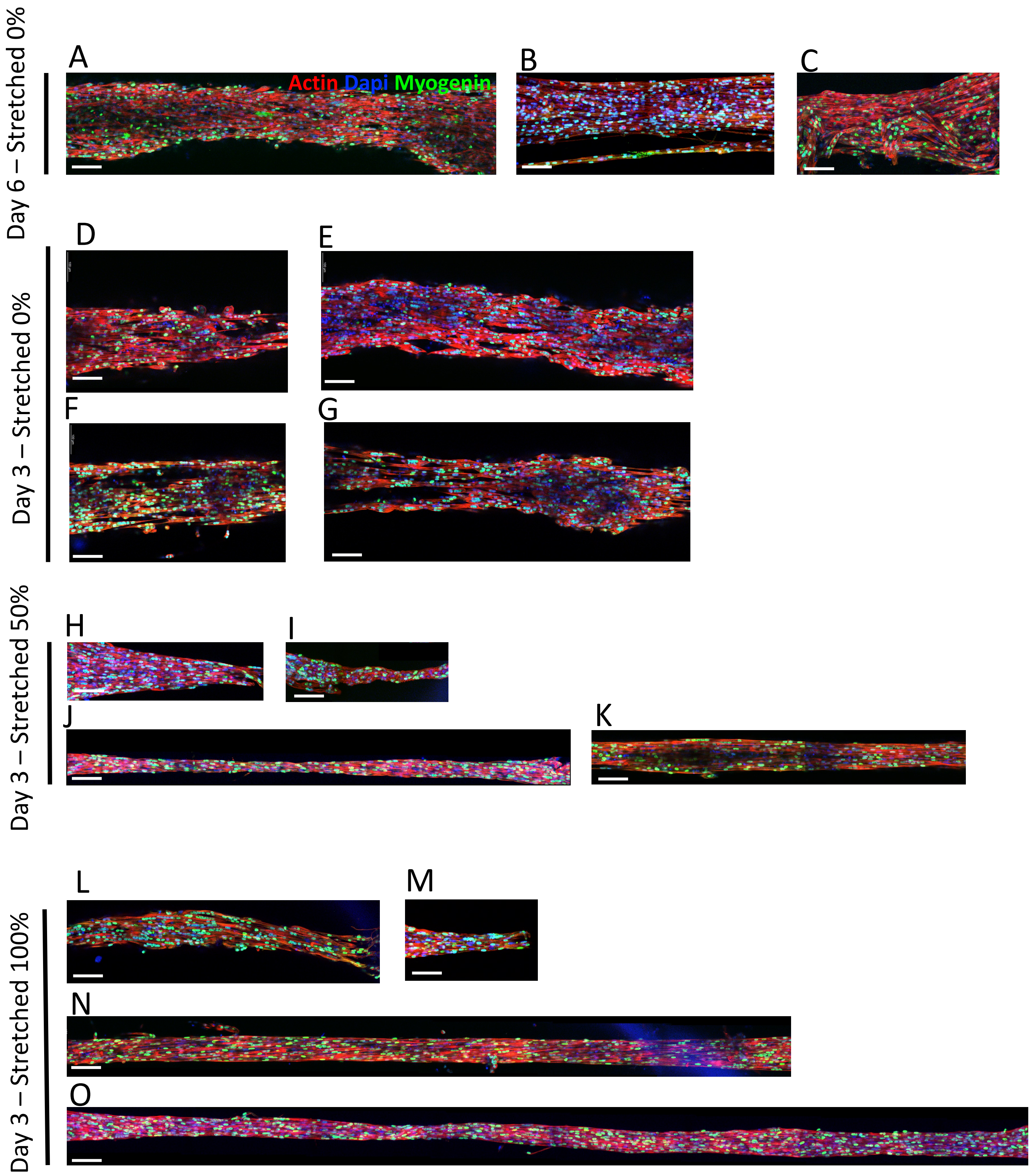

Fig. S27 . Confocal imaging of different myogenin immunostained tissues made from M-hiPSC 1 cultured for (A-C) 6 days with 0% stretching and 3 days with (D-G) 0%, (H-K) 50% and (L-O) 100% stretching. (actin in red, nuclei in blue, myogenin in green, scale bar=100µm)

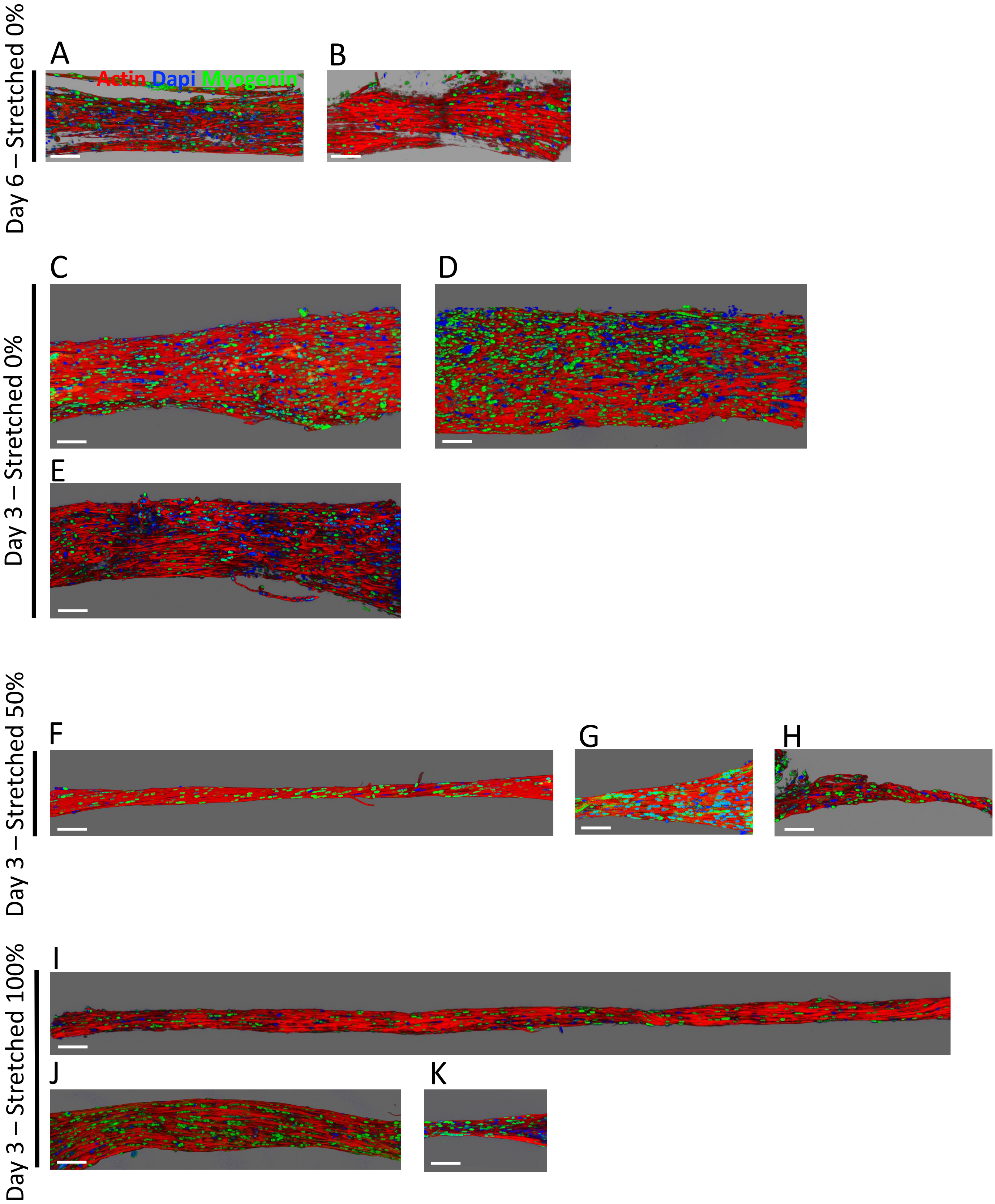

Fig. S28 . 3D reconstruction from confocal imaging of different myogenin immunostained tissues made from M-hiPSC 1 cultured for (A-B) 6 days with 0% stretching and 3 days with (C-E) 0%, (F-H) 50% and (I-K) 100% stretching. (actin in red, nuclei in blue, myogenin in green, scale bar=100µm)

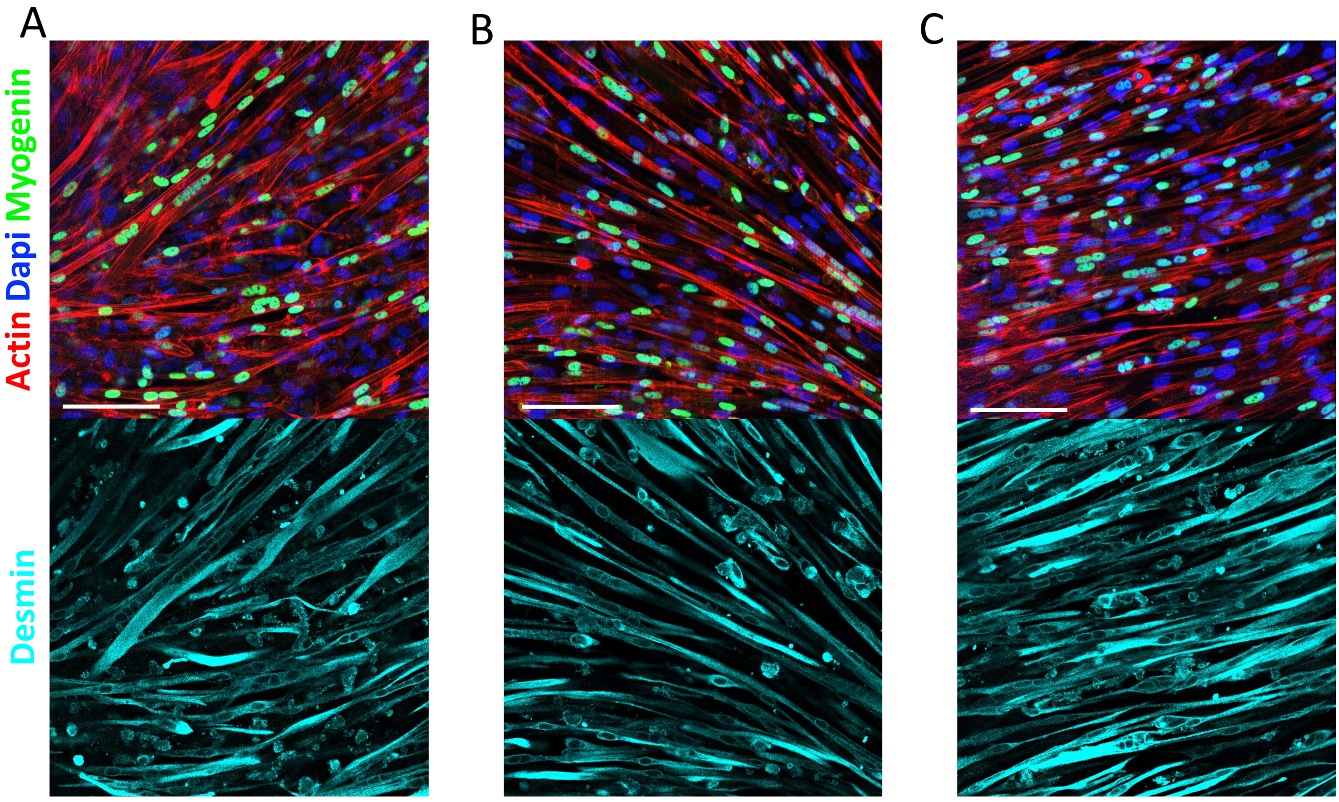

Fig. S29. Confocal imaging of three different samples of M-hiPSC 1 cultured for 6 days of differentiation in 2D, immunostained for myogenin and desmin (Actin in red, nuclei in blue, myogenin in green, desmin in cyan, scale bar = 100µm)

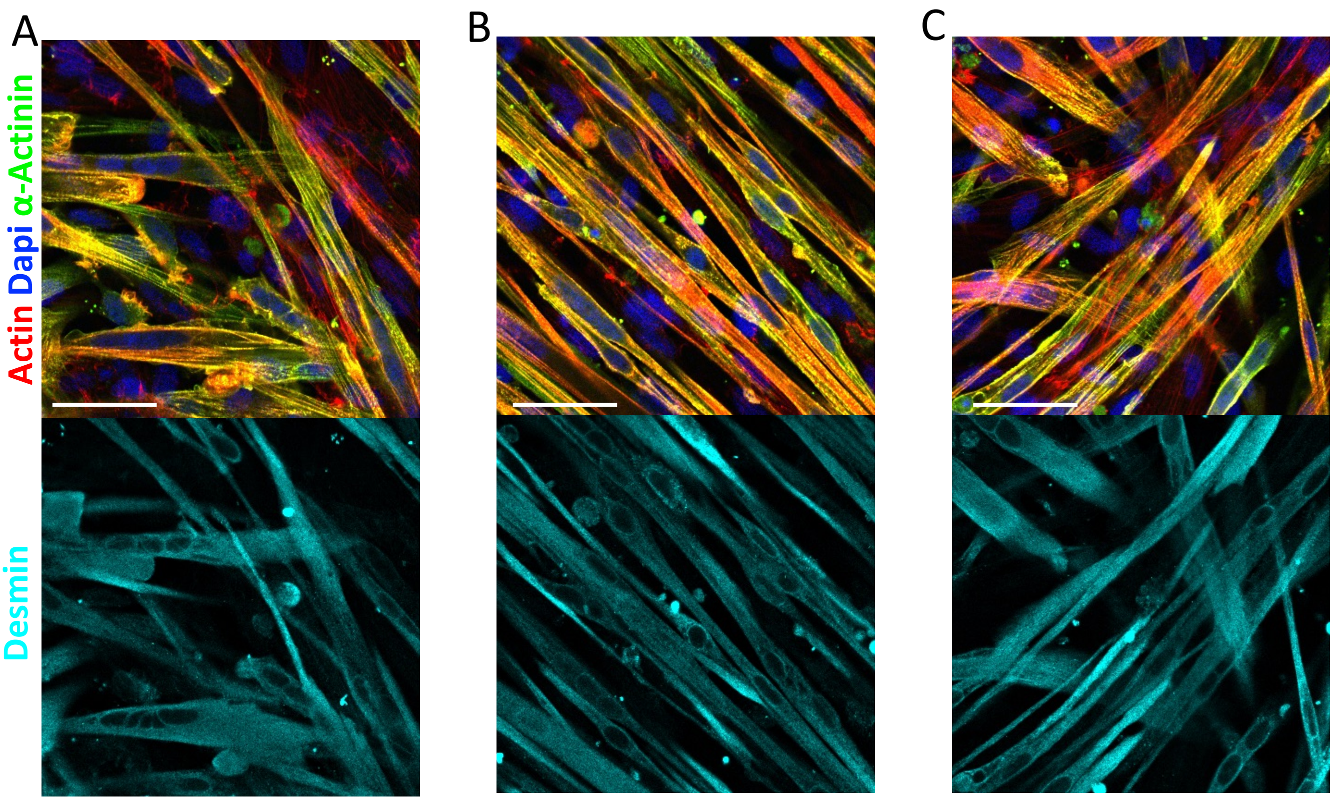

Fig. S30 . Confocal imaging of three different samples of M-hiPSC 1 cultured for 6 days of differentiation in 2D, immunostained for alpha-actinin and desmin (actin in red, nuclei in blue, alpha-actinin in green, desmin in cyan, scale bar = 50µm)

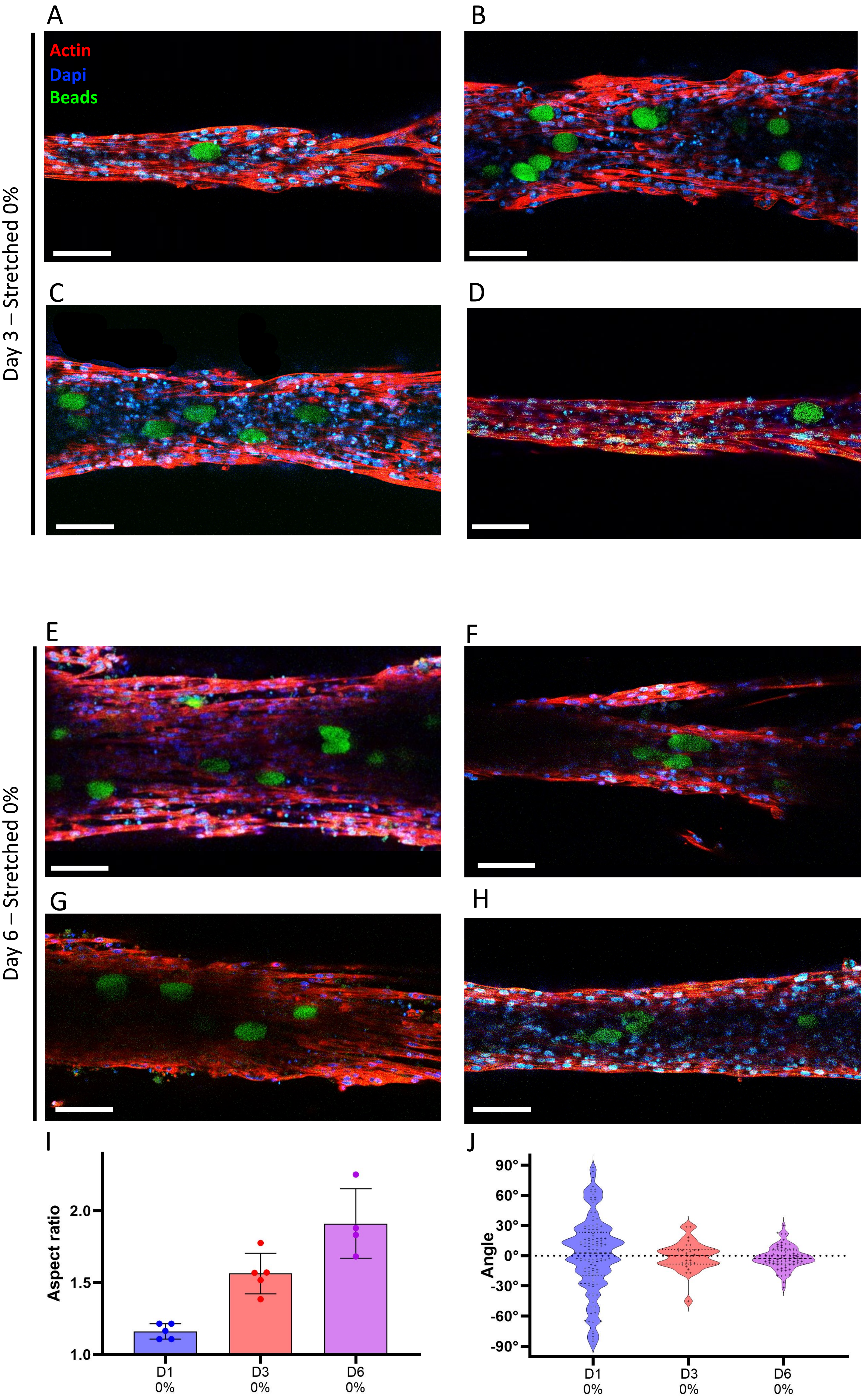

Fig. S31. Confocal imaging of different tissues made of M-hiPSC 1, in which polyacrylamide beads were embedded, which were cultured for (A-D) 3 days and (E-H) 6 days without stretching. (I) Aspect ratio and (J) orientation of the beads for tissues stretched by 0% on day 1, day 3 and day 6. Data points represent the average bead aspect ratio within one sample, and the angles of all beads across all samples. (Actin in red, Dapi in blue, beads in green, Scale bar = 100 µm)

Stretching improves functionality and specialization

A

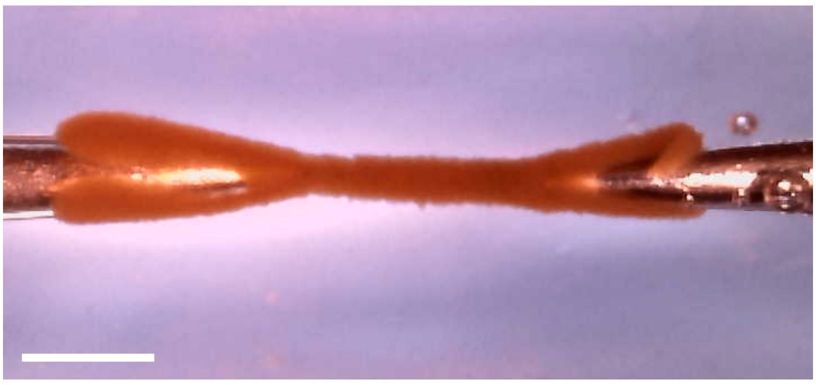

B

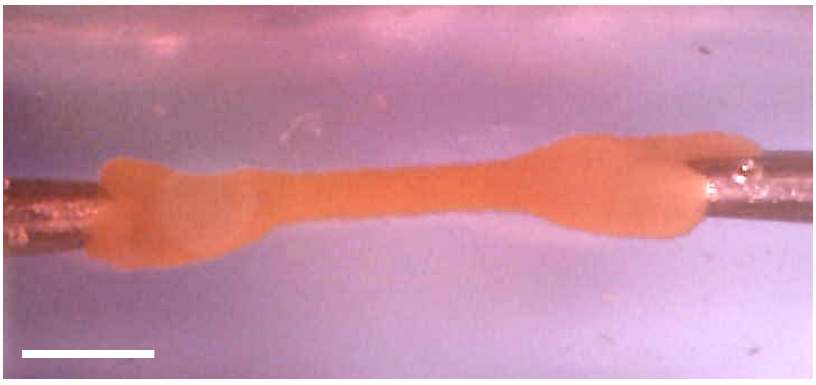

Fig. S32. Tissues magnetically bioprinted from magnetically labelled M-hiPSC 2 (A) and M-hiPSC 3 (B) trapped between needles. Scale bar=1mm

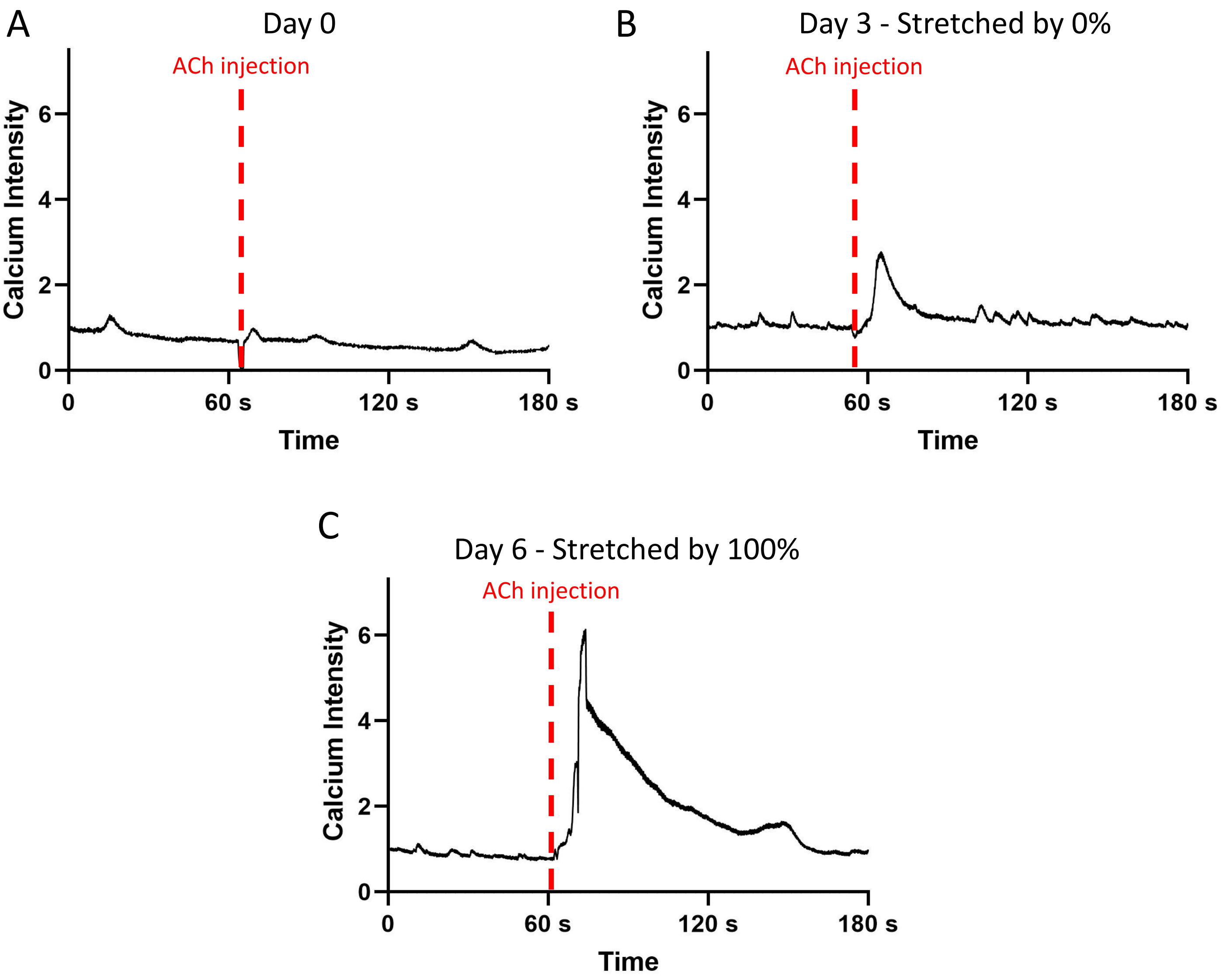

Fig. S33. Kymographs of the fluctuations of the calcium signal in a M-hiPS cell from (A) a M-hiPSC 2 tissue at day 0 just after bioprinting , (B) a non-stretched M-hiPSC 3 tissue at day 3, and (C) a M-hiPSC 2 tissue at day 3 stretched by 100% since day 1, treated with 1mM ACh after approximately 1min.

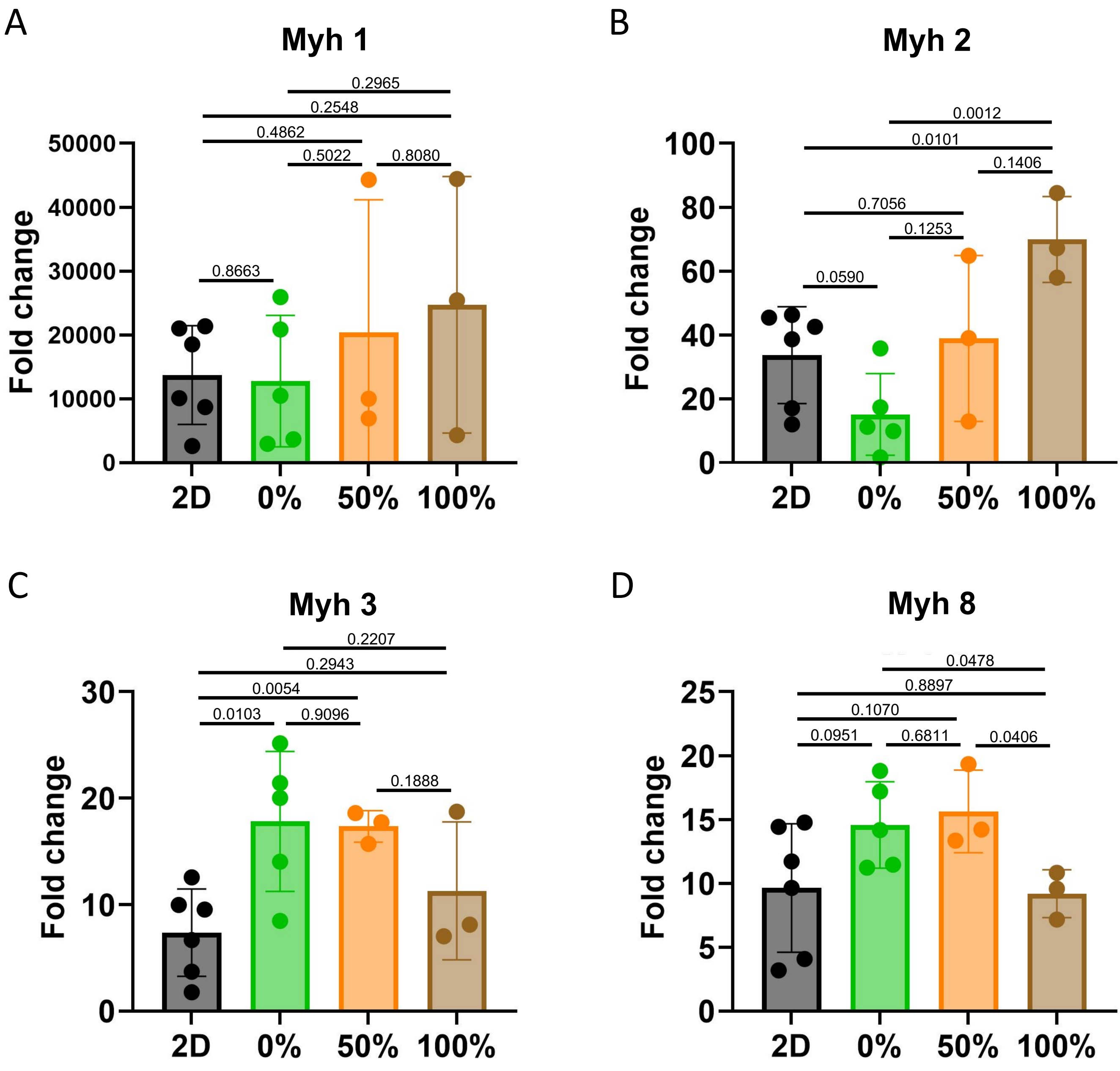

Fig. S34. Fold changes of the gene expression on day 3 of (A) Myh1, (B) Myh2, (C) Myh3 and (D) Myh8 for 3D tissues stretched by 0%, 50% and 100% and 2D cultures of M-hiPSC 1 , compared to the expression of cells collected at day 0.

Materials and Methods

	M-hiPSC 1	M-hiPSC 2	M-hiPSC 3
Sex of the patient	XX	XY	XX
Age of the patient	67	22	48
Origin of the sample	Fibroblasts	PBMC	PBMC 16525
Cell supplier	Phenocell		
Reprogramming method	Epi5 Kit - ThermoFischer #A15960		

Table S1. Information on the origin of the three patient derived hiPS cell lines from which the three M-hiPS cells were derived

F_18S	GAGGATGAGGTGGAACGTGT
R_18S	TCTTCAGTCGCTCCAGGTCT
F_Myh1	CTCTTTGTTGGGGCAACGG
R_Myh1	TGAGTGCTCCTCAAGTTGGTC
F_Myh2	TGAAGGAGAGGGAGCTGGT
R_Myh2	ATGGGTACTCCTGAGGTTGGT
F_MYH3	AGAGGAACTTTGACAAGGTGTTG
R_MYH3	GGCTTCCTCGTAGGCATTTT
F_Myh8	TGCTGAAGCAGATAGCAGCG
R_Myh8	TACGAAGTGAGGGTGTGTGC

Table S2. Primers used for qRT-PCR.
